# The Use of Non-targeted Lipidomics and Histopathology to Characterize the Neurotoxicity of Bifenthrin to Juvenile Rainbow Trout (*Oncorhynchus mykiss*)

**DOI:** 10.1021/acs.est.2c01542

**Published:** 2022-07-25

**Authors:** Jason T. Magnuson, Leslie Caceres, Nathan Sy, Chenyang Ji, Philip Tanabe, Jay Gan, Michael J. Lydy, Daniel Schlenk

**Affiliations:** † Department of Environmental Sciences, University of California, Riverside, California 92521, United States; ‡ College of Environment, Zhejiang University of Technology, Hangzhou 310032, China; § Department of Zoology, Center for Fisheries, Aquaculture and Aquatic Sciences, Southern Illinois University, Carbondale, Illinois 62901, United States; ∥ Institute of Environmental Health, College of Environmental and Resource Sciences, Zhejiang University, Hangzhou 310058, China

**Keywords:** pyrethroid, salmonid, neurotoxic, brain, histology, adverse outcome

## Abstract

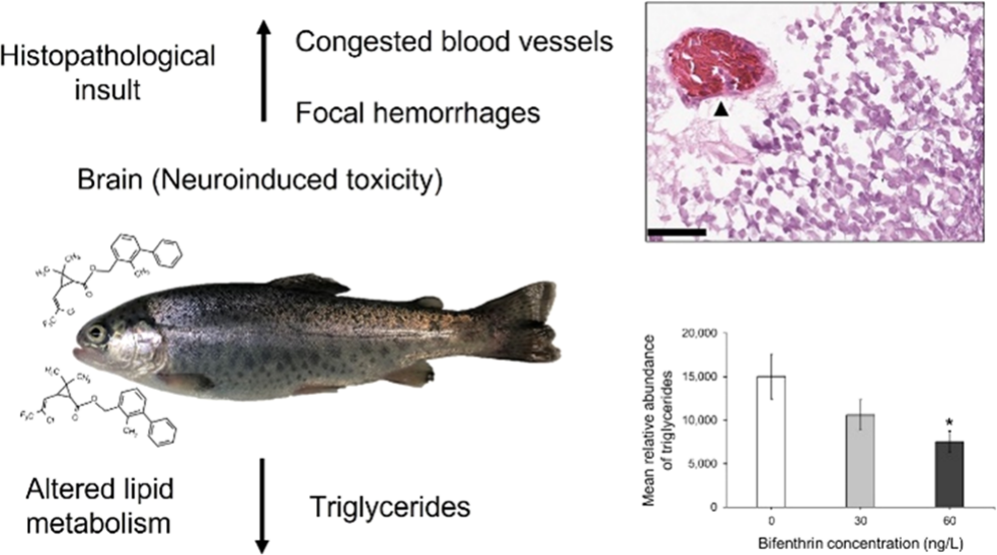

Due to the detection frequencies and measured concentrations in surface water, the type I pyrethroid insecticide, bifenthrin, has been of particular concern within the Sacramento-San Joaquin Delta in California. Concentrations have been detected above levels previously reported to impair neuroendocrine function and induce neurotoxicity to several species of salmonids. Metabolomic and transcriptomic studies indicated impairment of cellular signaling within the brain of exposed animals and potential alteration of lipid metabolism. To better understand the potential impacts of bifenthrin on brain lipids, juvenile rainbow trout (*Oncorhynchus mykiss*) were exposed to mean bifenthrin concentrations of 28 or 48 ng/L for 14 days, and non-targeted lipidomic profiling in the brain was conducted. Brain tissue sections were also assessed for histopathological insult following bifenthrin treatment. Bifenthrin-exposed trout had a concentration-dependent decrease in the relative abundance of triglycerides (TGs) with levels of phosphatidylcholines (PCs) and phosphatidylethanolamines (PEs) significantly altered following 48 ng/L bifenthrin exposure. An increased incidence of histopathological lesions, such as focal hemorrhages and congestion of blood vessels, was noted in the brains of bifenthrin-treated animals, suggesting an association between altered lipid metabolism and neuronal cell structure and integrity.

## Abstract 2

The brains of juvenile trout exposed to environmentally relevant bifenthrin concentrations had altered lipidomic profiles and histopathological insult.

## 1 Introduction

Newer-generation insecticides, such as pyrethroids, have reduced toxicity to mammals and lower environmental persistence than older-generation insecticides (organophosphates and organochlorines, respectively).^1^ Among the top used insecticides globally,^2^ pyrethroids may induce a greater environmental risk than organochlorines and organophosphates^3^ since they are the main class of current-use insecticides that exceed regulatory threshold levels to non-target aquatic organisms at several locations^4^ (regulatory threshold values of pyrethroids ranging from 0.850 to 265 ng/L).^3^ Among 32 insecticides that most frequently exceed regulatory threshold levels in surface waters and sediments, seven are pyrethroids, and bifenthrin, a type I pyrethroid, possessed an exceedance rate of 80.7% in more than 1800 samples analyzed throughout the United States.^5^

The global use of bifenthrin for pest control in India, Africa, Vietnam, Australia, China, and the United States has raised concerns about non-target species. Bifenthrin is the most frequently detected pyrethroid insecticide in sediment and surface water samples in California,^6^ with concentrations of bifenthrin detected in Australia and China similar to those reported in the United States, which were reported to induce a high frequency of invertebrate lethality.^2,7^ Concentrations of bifenthrin in California, particularly within the Sacramento-San Joaquin Delta (Delta), were present in 79% of samples collected, ranging from 0.2 to 133 ng/L.^8,9^ The Delta provides a spawning habitat and nursery for critically threatened and endangered fish species, such as steelhead trout (*Oncorhynchus mykiss*). Reductions in the number of anadromous fish species since the early 2000s have largely coincided with the increased use of pyrethroids, such as bifenthrin.^10−15^ The highest detection frequencies and concentrations of bifenthrin were found in water samples in predominately urban runoff locations and in stormwater runoff samples in the Delta at concentrations of 106 and 133 ng/L bifenthrin, respectively,^8,9^ and in some instances at concentrations exceeding 3 μg/L.^16^ Recently, it was reported that bifenthrin was detected in 100% of zooplankton samples collected in the Sacramento River, at concentrations of up to 691 ng/g lipid,^17^ a common prey item of juvenile salmonids in the Delta prior to outmigration.

Concentrations of bifenthrin previously measured in water samples collected from the Delta (15 ng/L to 1.50 μg/L) have been shown to alter neuroendocrine pathways, exhibit neurotoxicity through the dysregulation of transcriptomic and metabolomic pathways involved in apoptotic and inflammatory pathways, and induce histopathological effects on salmonids following exposure.^18−23^ Using transcriptomic and non-targeted metabolic approaches, we previously reported that the dysregulation of several genes and metabolites was associated with changes in neuronal signaling pathways by altering signaling fatty acids in bifenthrin-treated salmonids.^18−20^ However, lipidomic profiling in the brains of fish following pesticide treatment is limited,^24^ although it can help identify novel adverse outcome pathways (AOPs), relating molecular-level effects to higher levels of biological organization.

Lipidomics is a subset of metabolomics and is increasingly used as an endpoint to understand the effects of contaminants on lipid profiles,^24,25^ with non-targeted approaches considered the best when implemented in discovery-based studies.^25^ Lipids are involved in a broad spectrum of physiological functions that include structural support, trafficking, energy metabolism, and membrane signaling,^24^ to name a few. The integration of multiple omic techniques overlayed with lipidomic profiling data allows for a better characterization of mechanisms of toxicity,^24−26^ as, currently, the use of in silico software programs to annotate functional pathways with lipid species alone is lacking. The goal of this study was to use non-targeted lipidomic profiling to characterize classes of lipids that may be altered by bifenthrin in the brains of rainbow trout, a surrogate for the endangered California steelhead trout, and determine whether relationships to histopathological insult in this target organ exist. We hypothesized that bifenthrin would induce concentration-dependent alterations in lipid profiles in the brains of exposed trout that were previously proposed to be involved in lipid signaling pathways.

## 2 Materials and Methods

### 2.1 Experimental Design

Juvenile rainbow trout were obtained from Jess Ranch Hatchery (Apple Valley, CA) and maintained in a Living Stream (Frigid Units, Toledo, OH) at 12 °C for 4 months under a 14:10 h light/dark photoperiod prior to experimentation. Individual trout were placed in 8 L glass aquaria randomly, where they acclimated for 3 days prior to experimentation, as previously conducted.^19^ Juvenile trout (mean length = 17.73 ± 1.42 cm; mean weight = 41.28 ± 10.13 g) were exposed to a control (0.01% v/v ethanol (solvent), diluted with dechlorinated tap water), 30, or 60 ng/L bifenthrin (>98% purity, mix of isomers, Chem Service) for 14 days, as previously conducted.^19^ Concentrations and durations that caused histopathological damage in the brains of salmonids were selected based on previous studies.^20^ Each exposure treatment had eight replicates, with one fish per tank (*n* = 8 per exposure). Trout were fed every 48 h (1% body weight; Oncor Fry trout pellets, Skretting), and 50% static water changes were conducted daily to renew bifenthrin treatments. Following a 14 day exposure, trout were euthanized with an overdose of sodium bicarbonate-buffered MS-222 (Sigma-Aldrich, St. Louis, MO). Brains were extracted and either flash-frozen in liquid nitrogen and stored at −80 °C until lipidomic analysis or fixed in 4% paraformaldehyde for downstream histological analysis. This experiment was performed ethically and in accordance with the University of California, Riverside Institutional Animal Care and Use Committee (protocol no. 20130010).

### 2.2 Bifenthrin Chemistry Analysis

Samples of water were collected from two random tanks from each time point at three separate sampling events prior to water renewals, at the beginning (24 h), middle (7 days), and end (14 days) of the 14 day exposure and stored in 1 L glass amber bottles at 4 °C in the dark. All water samples were extracted within two weeks of collection and analyzed as previously described.^21^ Additionally, to determine the measured concentrations of bifenthrin in extracted water samples, a seven-point calibration curve was used. Decachlorobiphenyl was used as a recovery surrogate and added to each sample before extraction, as previously conducted.^18^

### 2.3 Lipidomics Sample Preparation

Individual rainbow trout brains (*n* = 4 per treatment) were weighed in 2 mL bead mill tubes on ice, and 1 mL of extraction solvent (6:3:1 methyl *tert*-butyl ether/methanol/water) was added per 136.6 mg of tissue, with a mean weight of 120.0 ± 11.5 mg (range: 103.1–132.7 mg). Samples were homogenized at 4 °C and then vortexed for 30 min at 4 °C. Next, 250 μL of water was added per 136.6 mg of tissue to induce phase separation. Samples were vortexed for 5 min at 4 °C and then centrifuged at 16,000*g* for 5 min at 4 °C. The top, nonpolar layer (100 μL) was transferred to a 2 mL glass vial and dried under a gentle stream of nitrogen. The dried residue was resuspended in 200 μL of 9:1 methanol/toluene and analyzed by liquid chromatography–mass spectrometry (LC–MS).

### 2.4 LC–MS Lipidomics

LC–MS lipidomics analysis was performed at the UC Riverside Metabolomics Core Facility as previously described,^27^ with minor modifications. Briefly, a Synapt G2-Si quadrupole time-of-flight MS (Waters) that was coupled to an I-class UPLC system (Waters) was used to perform analyses. A CSH C18 column (2.1 × 100 mm^2^, 1.7 μm) (Waters) was used to carry out separations. The mobile phases were (A) 60:40 acetonitrile/water with 10 mM ammonium formate and 0.1% formic acid and (B) 90:10 isopropanol/acetonitrile with 10 mM ammonium formate and 0.1% formic acid. The flow rate was 0.50 mL/min, and the column was held at 65 °C. The injection volume was 1 μL in positive ion mode and 3 μL in negative ion mode. The gradient is as follows: 0 min, 15% B; 2 min, 30% B; 3 min, 50% B; 10 min, 55% B; 14 min, 80% B; 16 min, 100% B; 20 min 100% B; and 20.5 min, 15% B.

The MS scan range was 50–1600 *m*/*z* with a 100 ms scan time. Source and desolvation temperatures were 150 and 600 °C, respectively. Desolvation gas was set to 1100 L/h, and cone gas was set to 150 L/h. All gases were nitrogen, except the collision gas, which was argon. The capillary voltage was 1 kV in positive ion mode and 2 kV in negative ion mode. A quality control sample, generated by pooling equal aliquots of each sample, was analyzed every 4–5 injections to monitor system stability and performance. Samples were analyzed in random order. Leucine enkephalin was infused and used for mass correction, as it is a well-known standard peptide in mass spectrometry for calibration.^28^

### 2.5 Data Processing and Lipidomics Analysis

Progenesis Qi software (Nonlinear Dynamics) was used for processing untargeted data (alignment, deconvolution, peak picking, normalization, integration, and spectral matching). Total ion abundance was used to normalize data. Features with a coefficient of variation (CV) greater than 30% or average abundance of less than 500 in the QC injections were removed.^29,30^ To aid in the identification of features that belonged to the same metabolite, RAMClust was used to assign features with a cluster ID, which enabled annotations to be made based on spectral matching.^31^ To assign an annotation confidence level, the standard initiative guidelines for metabolomics were used.^32,33^ Annotation level 1 indicates an MS and MS/MS match or MS and retention time match to an in-house database generated with authentic standards. Level 2a indicates an MS and MS/MS match to an external database. Level 2b indicates an MS and MS/MS match to the LipidBlast in silico database^34^ or an MS match and diagnostic evidence, such as the dominant presence of an *m*/*z* 85 fragment ion for acylcarnitines. Level 3 indicates an MS match, although some additional evidence is required, such as adducts are detected to sufficiently deduce the neutral mass or the retention time is in the expected region. Several mass spectral metabolite databases were searched against including Metlin,^35^ Mass Bank of North America,^36^ and an in-house database (University of California, Riverside Metabolomics Core).

### 2.6 Histopathological Analysis

Individual, whole brains (*n* = 4 from each treatment) were fixed in a 4% paraformaldehyde solution (1× phosphate-buffered saline (PBS); pH 7.4; VWR, Radnor, PA) overnight at 4 °C. Brains were rinsed three times with 1× PBS for 5 min each, cryoprotected in 35% sucrose (w/v) overnight at 4 °C, and placed in OTC where they were oriented and frozen at −80 °C. Samples were sent to HistoWiz (Brooklyn, NY) where they were sectioned (5 μm), stained (H&E stain), and imaged. Histopathological alterations were determined by quantifying the length of stratum marginale detached and the number of congested blood vessels and focal hemorrhages per millimeter.

### 2.7 Statistical Analysis

Levene’s test was used to assess the homogeneity of variance, and a Shapiro–Wilk test was used to assess normality. One-way analysis of variance (ANOVA) with Tukey’s posthoc test was used to compare mean differences in the relative abundance of lipid classes among treatment groups. IBM SPSS Statistics for Windows, version 24 (IBM Corp., Armonk, New York) was used for conducting one-way ANOVA statistical analyses. Statistical significance was determined if *p* < 0.05. Principle component analyses were performed using R (v.3.6.x).

## 3 Results

### 3.1 Bifenthrin Water Chemistry Analysis

Bifenthrin was not detected in control treatment groups. Mean bifenthrin concentrations ± standard deviation was 28.3 ± 6.0 ng/L bifenthrin in the nominal 30 ng/L treatment group and 48.0 ± 12.3 ng/L bifenthrin in the nominal 60 ng/L treatment group (Table S1). Mean percent PCB-209 surrogate recoveries were 94.3 ± 10.1, 90.3 ± 10.9, and 78.9 ± 18.1 in the control, 30, and 60 ng/L treatment groups, respectively.

### 3.2 Untargeted Lipidomic Profile Analysis

**Figure 1:**
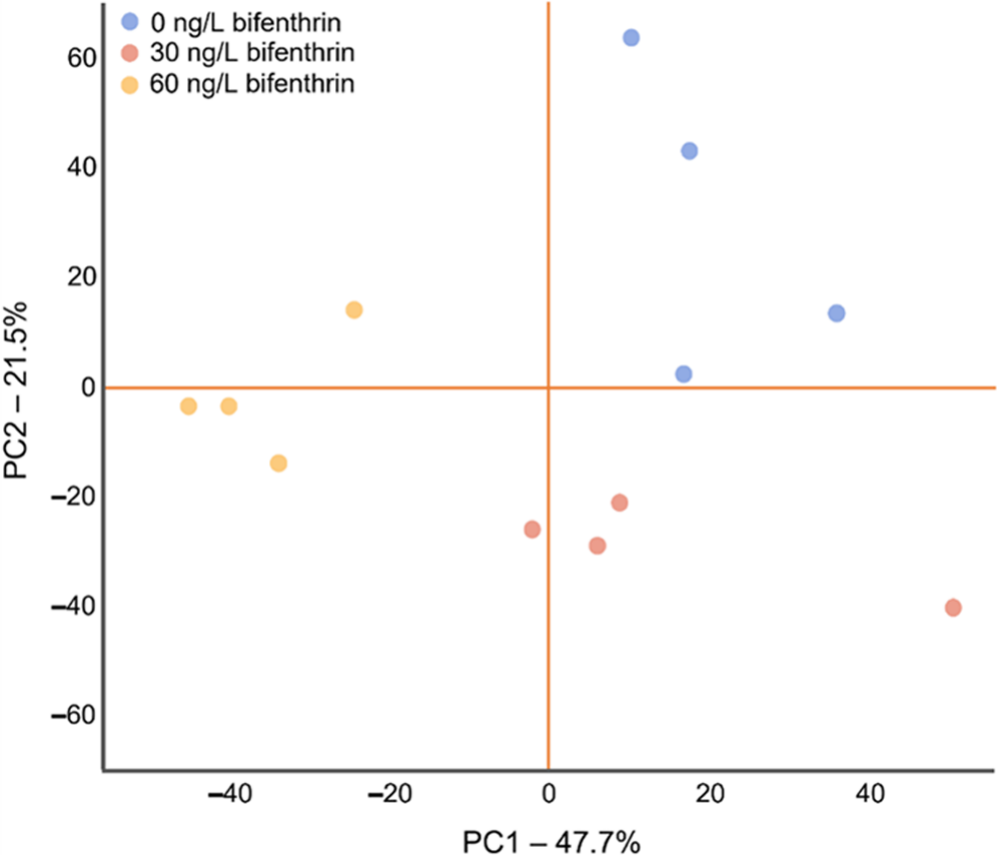
Principle component analysis (PCA) of the total lipid content in juvenile rainbow trout exposed to 0, 30, and 60 ng/L bifenthrin (*n* = 4 per treatment).

Principal component analyses indicated that all treatment groups were well separated from each other and strongly clustered based on the total lipid content (Figure 1). PC1 and PC2 explained 47.7% and 21.5% of the variance between treatment groups, respectively, with a total of 69.2% explained in the PCA score plot (Figure 1).

**Figure 2:**
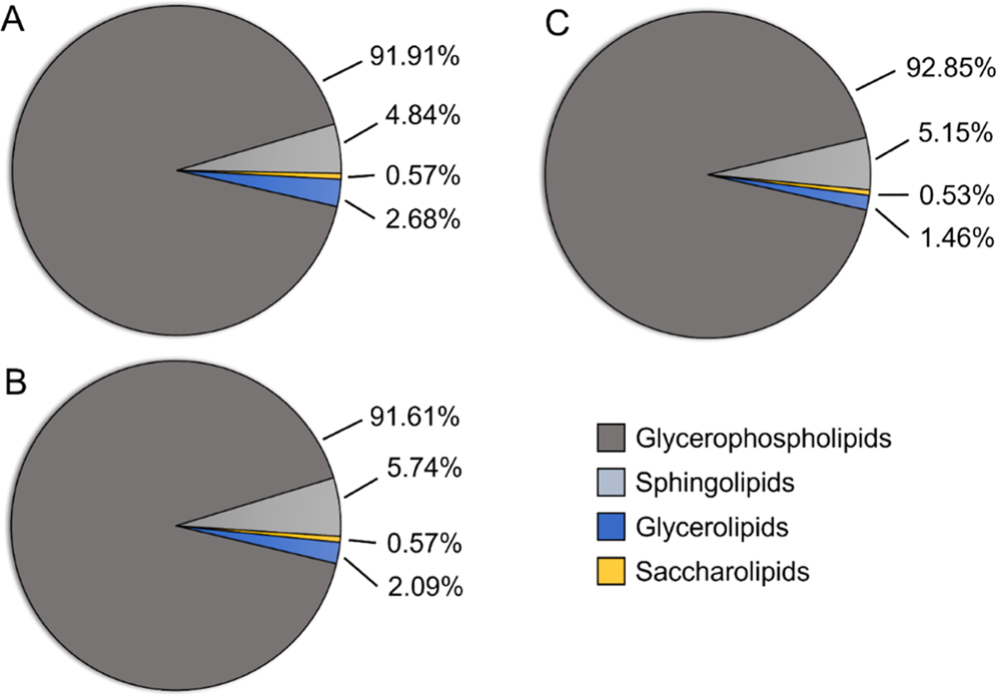
Relative percent abundance of the most representative lipids between (A) control, (B) 30, and (C) 60 ng/L bifenthrin treatment groups (*n* = 4 per treatment).

**Figure 3:**
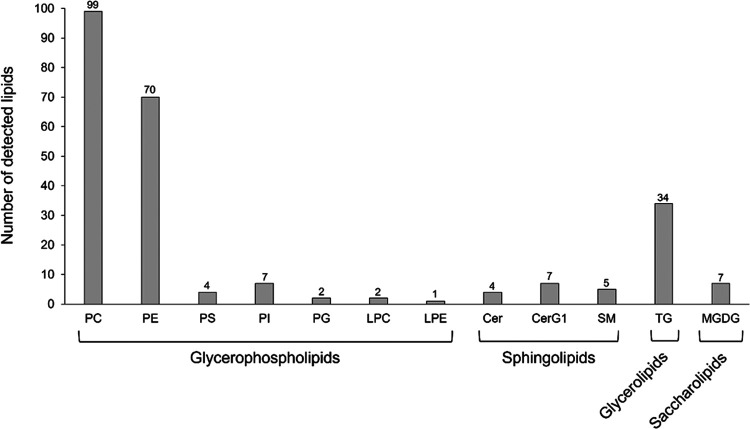
Number of detected lipids in lipidomic profiles. Phosphatidylcholine, PC; phosphatidylethanolamine, PE; phosphatidylserine, PS; phosphatidylinositol, PI; phosphatidylglycerol, PG; lysophosphatidylcholine, LPC; lysophosphatidylethanolamine, LPE; ceramide, Cer; monoglycosylceramide, G1Cer; sphingomyelin, SM; triglycerides, TG; and monogalactosyldiacylglycerol (MGDG) (*n* = 4 per treatment).

There were a total of four lipid classes (glycerolipids, glycerophospholipids, sphingolipids, and saccharolipids) representing 242 classified lipids in the brains of treated rainbow trout (Figure 2). Glycerolipids were represented by triglycerides (TGs); glycerophospholipids were represented by phosphatidylcholine (PC), phosphatidylethanolamine (PE), phosphatidylserine (PS), phosphatidylinositol (PI), phosphatidylglycerol (PG), lysophosphatidylcholine (LPC), and lysophosphatidylethanolamine (LPE); sphingolipids were represented by ceramide (Cer), monoglycosylceramide (G1Cer), and sphingomyelin (SM); and the saccharolipid was represented by monogalactosyldiacylglycerol (MGDG). The glycerophospholipid, PC, consisted of 99 different lipids, which were greater in number than TG > PE > PI, CerG1, MGDG > SM > SP, Cer > PG, LPC > LPE (Figure 3).

**Figure 4:**
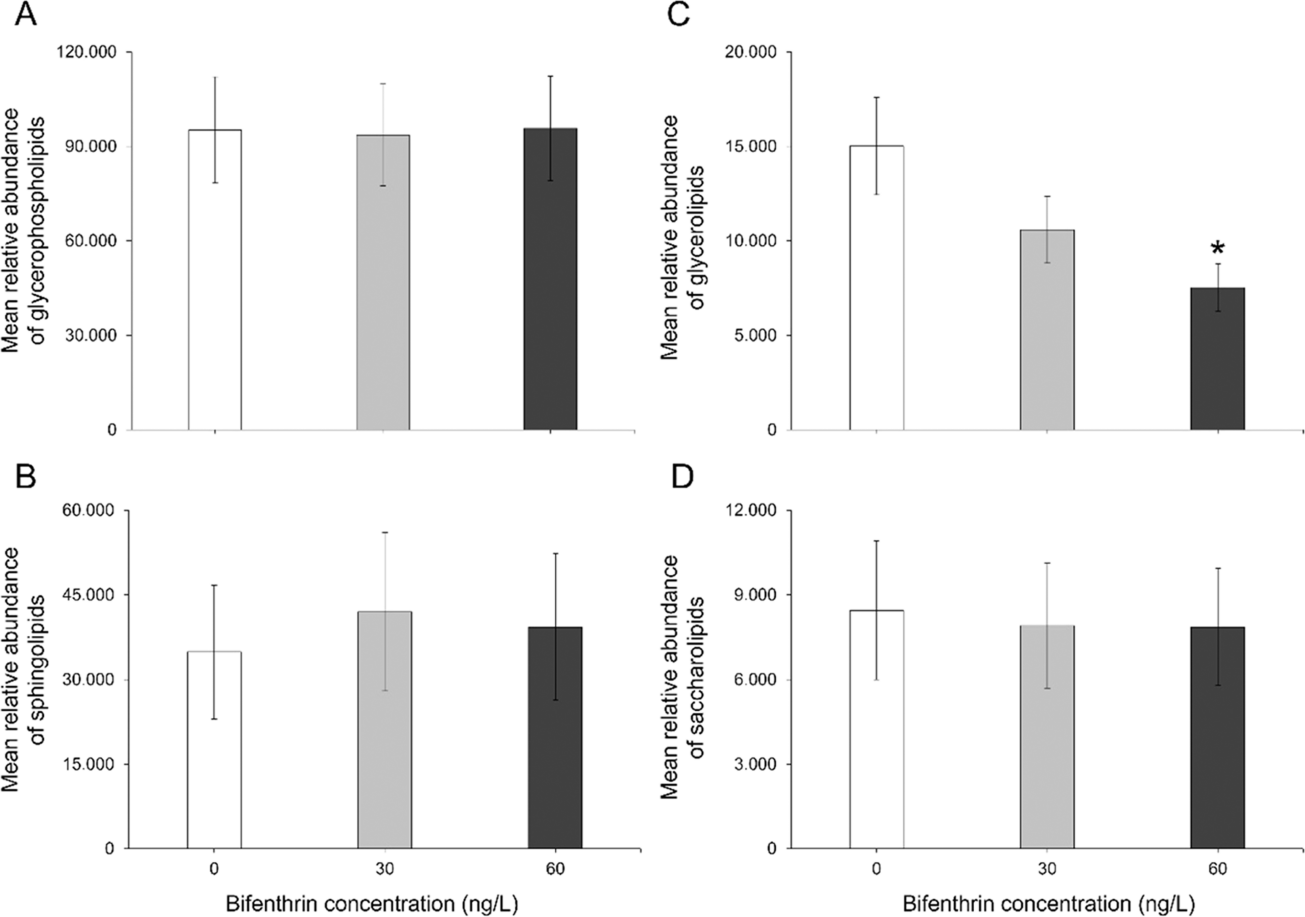
Mean relative abundance of (A) glycerophospholipids, (B) sphingolipids, (C) glycerolipids, and (D) saccharolipids in the brains of rainbow trout treated with 30 or 60 ng/L bifenthrin. Error bars represent SEM. Asterisks (*) denote statistical significance (*p* < 0.05) (one-way ANOVA and Tukey’s posthoc, *n* = 4 per treatment).

Glycerophospholipids had the largest relative abundance compared to sphingolipids, glycerolipids, and saccharolipids, and represented 91.91, 91.61, and 92.85% of total lipid abundance in control, 30, and 60 ng/L bifenthrin treatment groups, respectively. Sphingolipids had the second largest relative abundance, followed by glycerolipids and saccharolipids (Figure 2). There was a concentration-dependent decrease in the mean relative abundance of triglycerides between control and bifenthrin-treated fish (*p* = 0.036), although no significant difference was observed between the mean relative abundance of glycerophospholipids, sphingolipids, or saccharolipids, regardless of exposure treatment (*p* > 0.05; Figure 4).

Of the 99 identified PCs, 9 had a significantly increased abundance in the 60 ng/L bifenthrin treatment group, relative to controls (Figure S1). The significantly increased PCs were composed of the following lipids: PC 20:5_18:0, PC 30:1, PC 34:3, PC 36:3.1, PC 38:4.1, PC 38:6.1, PC 40:3, PC 42:3, and PC 44:4. Of the 70 identified PEs, 9 were significantly altered in the 60 ng/L bifenthrin treatment group, compared to control treatments. PEs that had a significantly increased abundance were composed of the following lipids: PE 20:5_18:2, PE 36:5, PE 38:7.1, PE 40:5, PE 42:10.1, PEP 32:1, PEP 36:3, and plasmenyl-PE 32:1. There was a single PE, PE 22:6_18:1, that was significantly decreased in the 60 ng/L bifenthrin treatment group (Figure S2). The relative lipid abundance between PS, PI, PG, LPC, and LPE was not significantly altered between treatment groups (Figures S3–S7).

The relative lipid abundance between Cer, G1Cer, and SM was not significantly altered between treatment groups (Figures S8–S10). Of the 34 identified TGs, 24 had a significant concentration-dependent decrease in relative abundance (Figure S11). The significantly reduced TGs were composed of the following lipids: TG 46:1, TG 48:1, TG 48:3, TG 50:2, TG 50:4, TG 51:2, TG 51:3, TG 52:3, TG 54:1, TG 54:2, TG 54:4, TG 54:5, TG 56:2, TG 56:3, TG 56:4, TG 56:5, TG 56:6, TG 56:7, TG 56:8, TG 58:10, TG 58:7, TG 58:8, TG 58:9, and TG 60:8 (Figure S11). The relative lipid abundance between MGDGs was not significantly altered between treatment groups (Figure S12).

### 3.3 Histopathological Characterization

**Figure 5:**
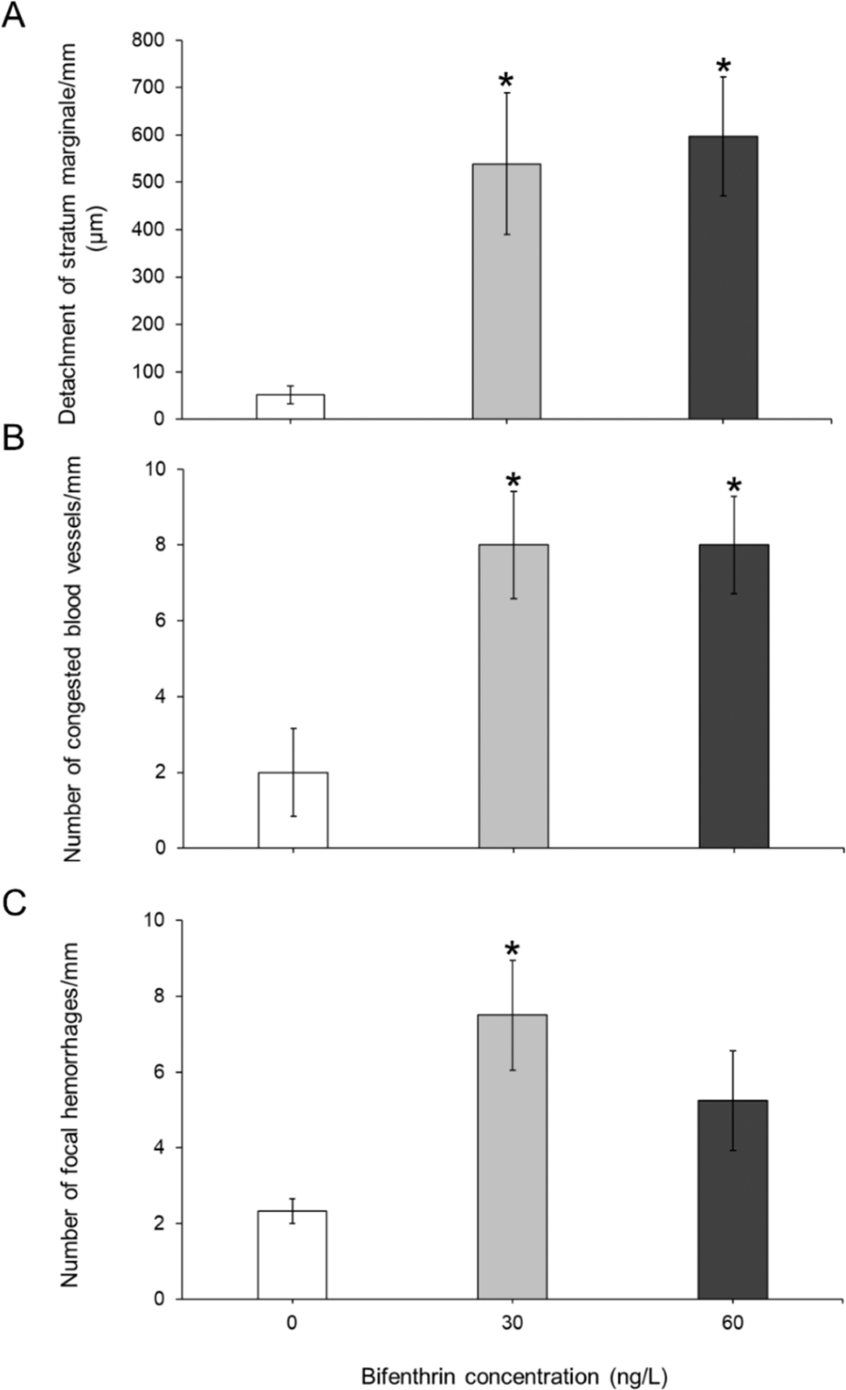
Incidence of (A) detached stratum marginale and the number of (B) congested blood vessels and (C) focal hemorrhages in the optic tectum of rainbow trout treated with 30 and 60 ng/L bifenthrin. Error bars represent SEM. Asterisks (*) denote statistical significance (*p* < 0.05) (one-way ANOVA and Tukey’s posthoc, *n* = 4 per treatment).

**Figure 6:**
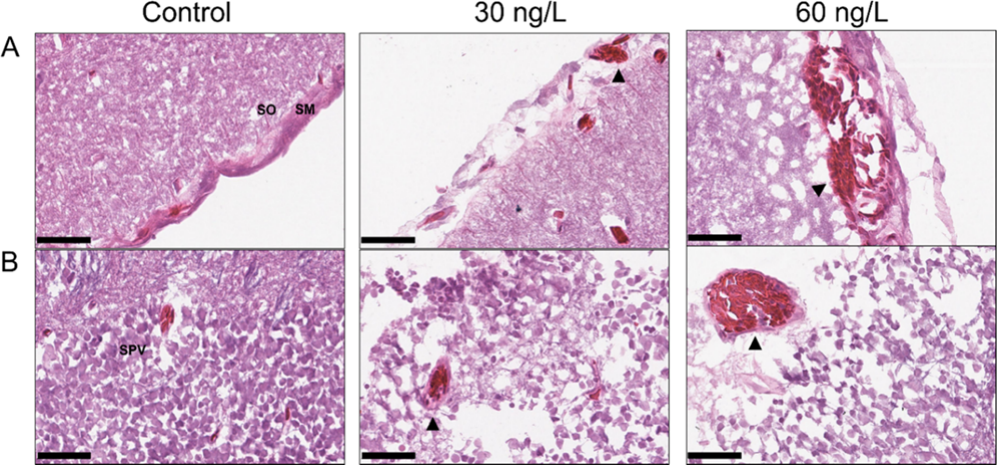
Histopathological characterization of structural alterations in the optic tectum of rainbow trout treated with 30 and 60 ng/L bifenthrin. (A) Detachment of the stratum marginale (SM) from the stratum opticum (SO) layer, denoted by a black arrow, and black triangles denote focal hemorrhages. (B) Dilation and congestion of blood vessels localized in the stratum periventriculare (SPV) denoted by black triangles. Black bars represent a 50 μm scale bar (400× magnification). H&E staining (*n* = 4 per treatment).

Juvenile rainbow trout exposed to 30 and 60 ng/L bifenthrin had a significantly increased incidence of a detached stratum marginale (Figures 5A and 6A; *p* = 0.041 and *p* = 0.015, respectively) and an increased number of congested blood vessels, relative to controls (Figures 5B and 6B; *p* = 0.036 and *p* = 0.036, respectively). The number of focal hemorrhages was significantly increased in trout treated with 30 ng/L bifenthrin but not in the 60 ng/L treatment (Figure 5C; *p* = 0.031 and *p* = 0.124, respectively). The mean areas of hemorrhages (Figure S13A) and congested blood vessels (Figure S13B) were not significantly different from those in control treatment groups, although these did have increasing trends with increased bifenthrin treatment (*p* > 0.05).

## 4 Discussion

Although previous studies have shown that contaminants can alter genes and metabolites involved in lipid metabolism and biosynthesis, the response of exposure to lipid pathways and alterations in lipid profiles is limited.^24^ Currently, there is a large gap in the field of ecotoxicology regarding the use of lipidomics to characterize the effects of contaminant exposure on lipids, which are largely involved in providing membrane fluidity, structural support, and energetics and can act as signaling molecules themselves. Advancements in analytical techniques have allowed for the identification and characterization of an increasing number of lipids,^37,38^ although current tools for constructing functional annotation pathways to understand the role and impact of altered levels of lipids are limited. The integration of multiple omic endpoints, such as transcriptomic and metabolomic profiling, overlayed with lipidomic data allows for a better representation of the underlying mechanisms following contaminant exposure to relate metabolic alterations to apical endpoints.^24^

Non-targeted lipidomic profiling was used to assess potential mechanisms responsible for bifenthrin’s neurotoxic response in salmonids following treatment to concentrations previously measured in the Delta (≤60 ng/L). There was a concentration-dependent decrease in the relative abundance of triglycerides in the brains of rainbow trout treated with bifenthrin after 2 weeks, with significant alterations in the abundance of several glycerophospholipids. Additionally, there was a subsequent increase in the incidence of histopathological insult in the brains of bifenthrin-treated fish noted by an increase in the detachment of the stratum marginale and the number of congested blood vessels and focal hemorrhages in the optic tectum.

Rainbow trout treated with bifenthrin had a significant reduction in the total abundance of glycerolipids, particularly TGs, representing 2.68% of the total lipid abundance in controls and 1.46% in trout treated with 60 ng/L bifenthrin, with a decrease of 71% of the identified TGs in bifenthrin-treated fish. Similar reductions of TGs were observed in the brains of rainbow trout treated with 1.38 μg/L chlorpyrifos.^39^ Atlantic salmon hepatocytes exposed to a mixture of chlorpyrifos-methyl, pirimiphos-methyl, and nonylphenol at a concentration of 100 μM also had a significantly reduced abundance of TGs.^40^ There was an upregulation in lipase (*lipe*) mRNA expression reported in chlorpyrifos-exposed hepatocytes, which has a prominent role in the hydrolysis of TGs.^41^ Additionally, the mRNA expression of *ppar*α was strongly correlated between fatty acid metabolites in hepatocytes of chlorpyrifos-exposed salmon, which was predicted to disrupt fatty acid β-oxidation,^42^ whereas *ppar*γ expression disrupted fatty acid oxidation in HepG2 cells treated with 1 × 10^–9^ to 1 × 10^–6^ M *cis*-bifenthrin, which was induced by the activation of pregnane X receptor (PXR) by bifenthrin.^43^ Reductions in the accumulation of TGs were among the top predicted alterations in chlorpyrifos-exposed salmon hepatocytes, with correlated oxidative stress responses predicted by metabolomic profiling and changes supported at the transcript level.^42^ Furthermore, the top predicted pathway in the livers of Atlantic salmon exposed to 8 mg/kg chlorpyrifos-methyl for 30 days was involved in regulating the concentration of TGs, which was driven by 16 differentially expressed genes.^44^ In contrast, HepG2 cells treated with either 50 μM cypermethrin, imidacloprid, or fipronil for 24 h had significantly elevated TG contents; however, 50 μM bifenthrin did not alter TG levels.^45^ A separate study found that when HepG2 cells were treated with 100 nM bifenthrin, there was a significant increase in TGs noted, and this effect was enantioselective.^43^

**Figure 7:**
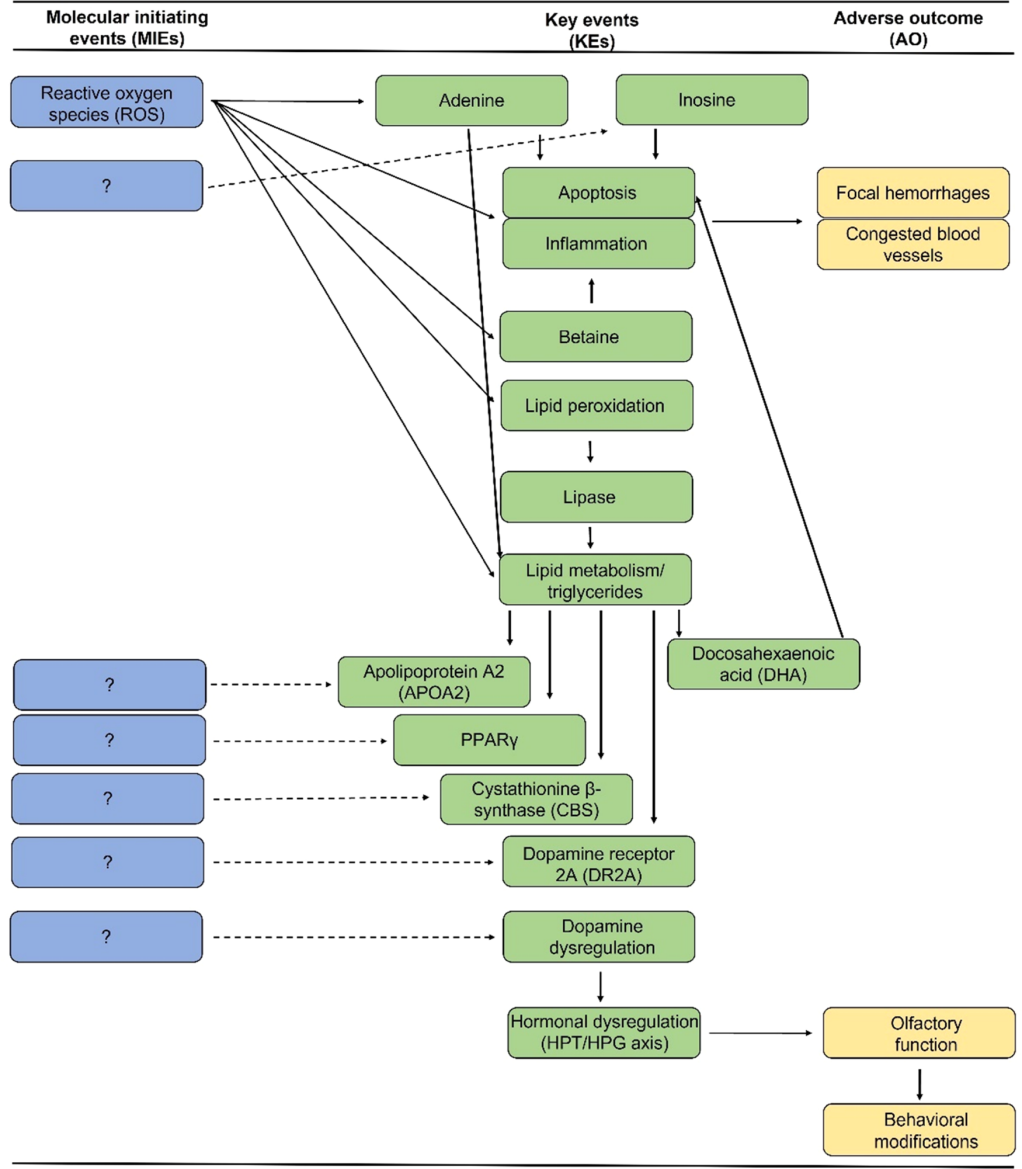
Hypothetical adverse outcome pathway (AOP) based on the integration of non-targeted metabolomic, transcriptomic, and lipidomic profiles with common targets of bifenthrin neurotoxicity in the brains of salmonids. Neuroendocrine effects are based on previously conducted salmonid studies and overlayed with predicted omic pathway analyses.

Previous studies with juvenile rainbow trout, steelhead trout, and Chinook salmon treated with bifenthrin at concentrations ranging between 15 ng/L and 1.50 μg/L showed altered metabolomic and transcriptomic profiles that predicted alterations in the metabolism of lipids in the brains of exposed fish.^18−20^ The metabolism of TGs, particularly, was the most predicted pathway to be impaired following bifenthrin treatment and was driven by decreased levels of docosahexaenoic acid (DHA); increased levels of betaine; and the altered expression of *apoa*2, *cbs*, and *lipe* (Figure 7). Lipoprotein lipase, a rate-limiting enzyme responsible for metabolizing TGs found in the brain, has a direct role in regulating the expression of dopamine receptor type 2 (*dr*2) in neurons^46^ and has been suggested to evoke a modulation in the dopaminergic system.^47^ Crago and Schlenk^23^ previously reported that the mRNA expression of dopamine receptor 2a (*dr*2*a*) was significantly decreased in the brains of juvenile rainbow trout treated with 0.15 and 1.5 μg/L bifenthrin for 96 h and 2 weeks, with concentration-dependent decreases found.^23^ Interestingly, Giroux et al.^22^ found that Chinook salmon alevin treated with 0.15 and 1.5 μg/L bifenthrin under three temperature regimes (11, 16.4, and 19 °C) had significant, concentration-dependent increases in *dr*2*a* mRNA expression at 11 °C, although a significant, concentration-dependent decrease in *dr*2*a* mRNA expression was noted at 16.4 °C.^22^ Expression profiles of *dr*2*a* may also be influenced by fish life stage and exposure temperature, as the significance of effects was correlated with temperature. The disruption of lipid metabolism, particularly TGs, is predicted to disrupt the dopaminergic system, suggesting that bifenthrin-induced neurotoxicity, and neuroendocrine effects along the dopaminergic pathway, may act through altered lipid pathways (Figure 7). Alterations to the dopaminergic pathway may further be related to alterations in olfactory function, as has been shown to be impaired in salmonids following bifenthrin exposure.^21^ Although the relationship between neurotoxicity-induced effects following bifenthrin treatment and neuroendocrine effects is not well understood, it can be suggested that altered levels of lipids in the brain may play a role in disrupting the dopaminergic system, possibly stemming from alterations in signaling lipids specifically or as a subsequent result of lipid metabolism and associated dopamine receptors.

PC and PE are the two most abundant membrane phospholipids^48,49^ and predominant lipids that comprise fish membranes.^50^ PCs are an important source for signaling molecules, such as diacylglycerol, phosphatidic acid, and arachidonic acid.^51−54^ Dysregulation of PC levels has been shown to induce alterations in cell structure, signal transduction, and apoptosis and is implicated in Alzheimer’s disease.^51−53,55,56^ The catabolism of acetylcholine produces choline, which can be incorporated into PC synthesis and then be converted to betaine.^57^ Increased levels of betaine can additionally increase the formation of PCs,^57^ and it has previously been reported that juvenile steelhead exposed to 120 ng/L bifenthrin had a significant increase in betaine levels in the brains of treated fish.^19^ Bifenthrin has also been shown to significantly decrease the activity and expression of acetylcholinesterase (AchE) in the brains of murine exposed to 0.6 and 2.1 mg/kg bifenthrin for 60 days.^58^ Similarly, grass carp exposed to 6 μg/L bifenthrin for 24, 48, 48, and 96 h noted significant decreases in AchE activity in fish brains with increased exposure duration,^59^ which may serve as a source of choline for the synthesis of PCs.

PEs are involved in important proinflammatory cell surface signaling processes.^60^ Increased levels of PE in neuronal membranes have been shown to increase lipid peroxidation in the brain and reduce the fluidity of membranes, which influences cell structure and integrity.^61^ There was a significant increase in several PEs and PCs in the brains of rainbow trout exposed to 60 ng/L bifenthrin. Atlantic salmon hepatocytes exposed to 100 μM chlorpyrifos-methyl for 48 h had significantly increased PC levels but had reductions in PE levels.^40^ However, when individual Atlantic salmon were dietarily treated with 0.1 and 1.0 mg/kg chlorpyrifos-methyl for 30 days, there were significant increases in PC levels observed in the liver. In contrast, decreased PC levels were found following 30 days of 8.0 mg/kg chlorpyrifos-methyl treatment and in 0.1 and 1.0 mg/kg treatments after a 67-day exposure.^62^ PE levels did not significantly differ in 0.1 and 1.0 mg/kg chlorpyrifos-methyl treatment groups exposed for 30 days but significantly decreased following a 67-day exposure.^62^ Chlorpyrifos treatments (1.38 μg/L) in rainbow trout for 7 days did not have significantly altered levels of PC in brain tissue, with only a slight decrease in PE levels being reported.^39^ An imbalance between levels of PE and PC in the brain is the major driver of motor neuron survival, which has implications for the incidence of neuronal disease^60^ and may be a contributing factor to bifenthrin-induced neurotoxicity, as previous studies have reported that other fishes residing within the Delta, such as inland silversides (*Menidia beryllina*) and Delta smelt (*Hypomesus transpacificus*), exposed to bifenthrin have induced neurobehavioral effects, which could potentially have population-level consequences.^63−65^ Alterations in PC and PE levels may be related to increased levels of fatty acids that are stored as TGs in lipid droplets within the cell, comprising a monolayer of phospholipids,^40^ and known to be present in higher numbers in vacuole membranes.^53^

An increase in the level of several PCs (PC 34:2; PC 38:4; and PC 38:5) and PEs (PE 36:5; PE 38:7; and PE 40:5) in the brains of bifenthrin-treated trout were also reported to have significantly increased concentrations in lipid rafts following altered DHA levels in a human T-cell line,^66^ which was previously shown to be one of the top metabolites altered in the brains of bifenthrin-treated steelhead.^19^ Lipid rafts have been shown to play an increasing role in signal transduction,^67^ inflammatory response,^68^ and mechanotransduction and repair of neuronal membranes following injury.^69^ Increased levels of PC and PE were suggested to have a direct effect on signaling proteins associated with lipid rafts, displacing the proteins when accumulating on the raft structure.^66^ Although lipid rafts were not separated from whole brains in the current lipidomic analysis, additional research is warranted to understand the specific role bifenthrin has in the potential disruption of signaling lipid rafts in the brain following treatment.

Consistent with altered cellular structures,^20^ bifenthrin-treated rainbow trout had an increased incidence of histopathological alterations. There was a significantly increased amount of stratum marginale detachment from the stratum opticum in the optic tectum and an increased number of congested blood vessels in trout treated with 30 and 60 ng/L bifenthrin with increased focal hemorrhages in the stratum opticum. It was previously shown that rainbow trout treated with 15 and 30 ng/L bifenthrin had an increase in the number of apoptotic cells in the cerebellum and optic tectum,^20^ which was related to impaired integrity of the extracellular matrix structure and was further supported by predicted apoptotic pathways following bifenthrin treatment in steelhead trout.^18^ In rat brains, a congestion of blood vessels was observed following treatments with a 3 mL/kg pyrethroid mixture (allethrin, imiprothrin, and phenothrin) for 40 days.^70^ Mahseer (*Tor putitora*) exposed to 63 μg/L cypermethrin for 96 h^71^ and silver carp (*Hypophthalmichthys molitrix*) exposed to 2 μg/L deltamethrin for 96 h^72^ exhibited infiltration, spongiosis, and neuronal degeneration in brain tissues. Rats administered with 50 mM cypermethrin exhibited brain congestion when examined histologically, which correlated with increased neuronal lipid peroxidation.^73^ This suggests that a shared mechanism among pyrethroids may occur, causing specific histopathological effects in the brain, which may be related to lipid metabolism pathways that impair cellular structure and integrity.

The altered levels of several metabolites and dysregulated genes in the brains of juvenile salmonids following bifenthrin treatment were predicted to impair common pathways involved in lipid metabolism due to an increased incidence of oxidative stress that resulted in an induction of reactive oxygen species and subsequent downstream events that can be linked to non-targeted metabolomic, transcriptomic, and lipidomic analyses (Figure 7). The induction of apoptosis, inflammation, and lipid peroxidation may be directly due to increased reactive oxygen species in the brains of bifenthrin-treated salmonids.^18,19^ Apoptotic responses were the top predicted pathways in the brains of steelhead due to reduced levels of DHA, inosine, and adenine.^19^ Adverse effects were noted with an increased incidence of TUNEL-positive cells in the brain of bifenthrin-treated steelhead, with additional significant histopathological insult noted by increased focal hemorrhages and congested blood vessels. Induced inflammatory responses were predicted to be related to an increase in betaine levels, which may be due to disruptions in the metabolism of acetylcholine^57^ where levels were previously reported to be altered in the brains of carp exposed to bifenthrin,^59^ altering levels of choline available for phosphatidylcholine synthesis.

The use of a hypothetical AOP to collectively synthesize the alterations of transcriptomic and non-targeted metabolomic and lipidomic profiles is useful to conceptualize neurotoxic and neuroendocrine effects in salmonids following bifenthrin exposure (Figure 7). The induction of oxidative stress following bifenthrin exposure, noted by alterations in the production of reactive oxygen species and subsequent incidence of apoptosis, inflammation, and lipid peroxidation, may be a result of altered levels of structural and storage lipids that can be linked to the increased histopathological insult. Additionally, altered concentrations of key signaling lipids may be influenced by lipoprotein lipase in the brain, targeting TGs and potentially resulting in an impact to the dopaminergic system through alterations in dopamine receptor expression, which could influence olfactory behavior. Although additional, targeted studies are needed to explore this hypothetical AOP based on non-targeted analyses and relationships to previously identified neuroendocrine endpoints, this serves as a basis of future research to better understand more targeted mechanisms of bifenthrin in the brain of fish. The predominant focus of the integrated overlay of previously conducted metabolomic and transcriptomic studies with lipidomics was based on a single family of fish, salmonids, as a great amount of data is required that has similar exposure profiles to limit the influence of additional variables that may impact bifenthrin toxicity. However, alternative exposure routes should be considered in future studies, as well as other threatened and endangered fish in the Delta, as pesticide-laden diets have recently been reported to impair swimming performance in inland silversides and Chinook salmon,^74−76^ with predominate targets involved in upstream lipid metabolism and energetic pathways. The framework of comparing multiple omic profiles to understand the underlying mechanistic effects of contaminants, while considering subsequent species and pesticide exposure routes, is warranted and will allow for more informative decisions to be made and results implemented in future risk assessments.

## Supporting Information Available

Mean bifenthrin concentrations (Table S1), mean relative abundance of lipid classes (Figures S1–S12), and mean area of focal hemorrhages and congestion of blood vessels (Figure S13) (PDF)

## References

[ref1] Bradbury S. P.; Coats J. R. Comparative Toxicology of the Pyrethroid Insecticides. In Reviews of Environmental Contamination and Toxicology; Ware G. W., Ed.; Springer: New York, 1989; Vol. 108, pp 133–177. 10.1007/978-1-4613-8850-0_4 2646661

[ref2] Li H.; Cheng F.; Wei Y.; Lydy M. J.; You J. Global Occurrence of Pyrethroid Insecticides in Sediment and the Associated Toxicological Effects on Benthic Invertebrates: An Overview. J. Hazard. Mater. 2017, 324, 258–271. 10.1016/j.jhazmat.2016.10.056. 27825741

[ref3] Stehle S.; Schulz R. Agricultural Insecticides Threaten Surface Waters at the Global Scale. Proc. Natl. Acad. Sci. U.S.A. 2015, 112, 5750–5755. 10.1073/pnas.1500232112. 25870271 PMC4426442

[ref4] Schulz R.; Bub S.; Petschick L. L.; Stehle S.; Wolfram J. Applied Pesticide Toxicity Shifts toward Plants and Invertebrates, Even in GM Crops. Science 2021, 372, 81–84. 10.1126/science.abe1148. 33795455

[ref5] Wolfram J.; Stehle S.; Bub S.; Petschick L. L.; Schulz R. Meta-Analysis of Insecticides in United States Surface Waters: Status and Future Implications. Environ. Sci. Technol. 2018, 52, 14452–14460. 10.1021/acs.est.8b04651. 30472849

[ref6] Fuller N.; Anzalone S. E.; Huff Hartz K. E.; Whitledge G. W.; Acuña S.; Magnuson J. T.; Schlenk D.; Lydy M. J. Bioavailability of Legacy and Current-Use Pesticides in Juvenile Chinook Salmon Habitat of the Sacramento River Watershed: Importance of Sediment Characteristics and Extraction Techniques. Chemosphere 2022, 298, 134174 10.1016/j.chemosphere.2022.134174. 35276115

[ref7] Tang W.; Wang D.; Wang J.; Wu Z.; Li L.; Huang M.; Xu S.; Yan D. Pyrethroid Pesticide Residues in the Global Environment: An Overview. Chemosphere 2018, 191, 990–1007. 10.1016/j.chemosphere.2017.10.115. 29145144

[ref8] Sanders C. J.; Orlando J. L.; Hladik M. L. Detections of Current-Use Pesticides at 12 Surface Water Sites in California during a 2-Year Period Beginning in 2015: U.S. Geological Survey Data Series 1088; U.S. Geological Survey: Reston, VA, 2018; p 40.

[ref9] Weston D. P.; Lydy M. J. Urban and Agricultural Sources of Pyrethroid Insecticides to the Sacramento-San Joaquin Delta of California. Environ. Sci. Technol. 2010, 44, 1833–1840. 10.1021/es9035573. 20121184

[ref10] Brooks M. L.; Fleishman E.; Brown L. R.; Lehman P. W.; Werner I.; Scholz N.; Mitchelmore C.; Lovvorn J. R.; Johnson M. L.; Schlenk D.; Van Drunick S.; Drever J. I.; Stoms D. M.; Parker A. E.; Dugdale R. Life Histories, Salinity Zones, and Sublethal Contributions of Contaminants to Pelagic Fish Declines Illustrated with a Case Study of San Francisco Estuary, California, USA. Estuaries Coasts 2012, 35, 603–621. 10.1007/s12237-011-9459-6.

[ref11] Jorgenson B.; Brown L.; Fleishman E.; Macneale K.; Schlenk D.; Spromberg J.; Werner I.; Weston D.; Young T. M.; Zhang M.; Zhao Q. Predicted Transport Of Pyrethroid Insecticides From An Urban Landscape To Surface Water. Environ. Toxicol. Chem. 2013, 32, 2469–2477. 10.1002/etc.2352. 24115122 PMC3949623

[ref12] Katz J.; Moyle P. B.; Quiñones R. M.; Israel J.; Purdy S. Impending Extinction of Salmon, Steelhead, and Trout (Salmonidae) in California. Environ. Biol. Fishes 2013, 96, 1169–1186. 10.1007/s10641-012-9974-8.

[ref13] Mills T. J.; McEwan D. R.; Jennings M. R. California Salmon and Steelhead: Beyond the Crossroads. In Pacific Salmon and Their Ecosytems; Stouder D. J.; Bisson P. A.; Naiman R. J., Eds.; Chapman and Hall: New York, 1997.

[ref14] Sommer T.; Armor C.; Baxter R.; Breuer R.; Brown L.; Chotkowski M.; Culberson S.; Feyrer F.; Gingras M.; Herbold B.; Kimmerer W.; Mueller-solger A.; Nobriga M.; Souza K. The Collapse of Pelagic Fishes in the Upper San Francisco Estuary: El Colapso de Los Peces Pelagicos En La Cabecera Del Estuario San Francisco. Fisheries 2007, 32, 270–277. 10.1577/1548-8446(2007)32[270:TCOPFI]2.0.CO;2.

[ref15] Weston D. P.; Schlenk D.; Riar N.; Lydy M. J.; Brooks M. L. Effects of Pyrethroid Insecticides in Urban Runoff on Chinook Salmon, Steelhead Trout, and Their Invertebrate Prey. Environ. Toxicol. Chem. 2015, 34, 649–657. 10.1002/etc.2850. 25545717

[ref16] Siepmann S.; Holm S. Hazard Assessment of the Synthetic Pyrethroid Insecticides Bifenthrin, Cypermethrin, Esfenvalerate, and Permethrin to Aquatic Organisms in the Sacramento-San Joaquin River System. Office of Spill Prevention and Response 00-6, Administrative Report; California Department of Fish and Game: Sacramento, CA, USA; 2000.

[ref17] Anzalone S. E.; Fuller N. W.; Huff Hartz K. E.; Fulton C. A.; Whitledge G. W.; Magnuson J. T.; Schlenk D.; Acuña S.; Lydy M. J. Pesticide Residues in Juvenile Chinook Salmon and Prey Items of the Sacramento River Watershed, California – A Comparison of Riverine and Floodplain Habitats. Environ. Pollut. 2022, 303, 119102 10.1016/j.envpol.2022.119102. 35257807

[ref18] Magnuson J. T.; Giroux M.; Cryder Z.; Gan J.; Schlenk D. The Use of Non-Targeted Metabolomics to Assess the Toxicity of Bifenthrin to Juvenile Chinook Salmon (*Oncorhynchus tshawytscha*). Aquat. Toxicol. 2020, 224, 105518 10.1016/j.aquatox.2020.105518. 32474292

[ref19] Magnuson J. T.; Cryder Z.; Andrzejczyk N. E.; Harraka G.; Wolf D. C.; Gan J.; Schlenk D. Metabolomic Profiles in the Brains of Juvenile Steelhead (*Oncorhynchus mykiss*) Following Bifenthrin Treatment. Environ. Sci. Technol. 2020, 54, 12245–12253. 10.1021/acs.est.0c04847. 32900186

[ref20] Magnuson J. T.; Huff Hartz K. E.; Fulton C. A.; Lydy M. J.; Schlenk D. Transcriptomic and Histopathological Effects of Bifenthrin to the Brain of Juvenile Rainbow Trout (*Oncorhynchus mykiss*). Toxics 2021, 9, 48 10.3390/toxics9030048. 33807887 PMC8000926

[ref21] Giroux M.; Vliet S. M. F.; Volz D. C.; Gan J.; Schlenk D. Mechanisms behind Interactive Effects of Temperature and Bifenthrin on the Predator Avoidance Behaviors in Parr of Chinook Salmon (*Oncorhynchus tshawytscha*). Aquat. Toxicol. 2019, 216, 105312 10.1016/j.aquatox.2019.105312. 31563086

[ref22] Giroux M.; Gan J.; Schlenk D. The Effects of Bifenthrin and Temperature on the Endocrinology of Juvenile Chinook Salmon. Environ. Toxicol. Chem. 2019, 38, 852–861. 10.1002/etc.4372. 30681194

[ref23] Crago J.; Schlenk D. The Effect of Bifenthrin on the Dopaminergic Pathway in Juvenile Rainbow Trout (*Oncorhynchus Mykiss*). Aquat. Toxicol. 2015, 162, 66–72. 10.1016/j.aquatox.2015.03.005. 25781393

[ref24] Dreier D. A.; Bowden J. A.; Aristizabal-Henao J. J.; Denslow N. D.; Martyniuk C. J. Ecotoxico-Lipidomics: An Emerging Concept to Understand Chemical- Metabolic Relationships in Comparative Fish Models. Comp. Biochem. Physiol., Part D: Genomics Proteomics 2020, 36, 100742 10.1016/j.cbd.2020.100742. 32956922 PMC7669741

[ref25] Aristizabal-Henao J. J.; Ahmadireskety A.; Griffin E. K.; Ferreira Da Silva B.; Bowden J. A. Lipidomics and Environmental Toxicology: Recent Trends. Curr. Opin. Environ. Sci. Health 2020, 15, 26–31. 10.1016/j.coesh.2020.04.004.

[ref26] Koelmel J. P.; Napolitano M. P.; Ulmer C. Z.; Vasiliou V.; Garrett T. J.; Yost R. A.; Prasad M. N. V.; Godri Pollitt K. J.; Bowden J. A. Environmental Lipidomics: Understanding the Response of Organisms and Ecosystems to a Changing World. Metabolomics 2020, 16, 56 10.1007/s11306-020-01665-3. 32307636

[ref27] Reddam A.; Mitchell C. A.; Dasgupta S.; Kirkwood J. S.; Vollaro A.; Hur M.; Volz D. C. MRNA-Sequencing Identifies Liver as a Potential Target Organ for Triphenyl Phosphate in Embryonic Zebrafish. Toxicol. Sci. 2019, 172, 51–62. 10.1093/toxsci/kfz169. PMC6813745 31368501

[ref28] Sztáray J.; Memboeuf A.; Drahos L.; Vékey K. *Leucine enkephalin* - A Mass Spectrometry Standard. Mass Spectrom. Rev. 2011, 30, 298–320. 10.1002/mas.20279. 20669325

[ref29] Dunn W. B.; Broadhurst D.; Begley P.; Zelena E.; Francis-Mcintyre S.; Anderson N.; Brown M.; Knowles J. D.; Halsall A.; Haselden J. N.; Nicholls A. W.; Wilson I. D.; Kell D. B.; Goodacre R. Procedures for Large-Scale Metabolic Profiling of Serum and Plasma Using Gas Chromatography and Liquid Chromatography Coupled to Mass Spectrometry. Nat. Protoc. 2011, 6, 1060–1083. 10.1038/nprot.2011.335. 21720319

[ref30] Barupal D. K.; Fan S.; Wancewicz B.; Cajka T.; Sa M.; Showalter M. R.; Baillie R.; Tenenbaum J. D.; Louie G.; Kaddurah-Daouk R.; Fiehn O. Generation and Quality Control of Lipidomics Data for the Alzheimer’s Disease Neuroimaging Initiative Cohort. Sci. Data 2018, 5, 180263 10.1038/sdata.2018.263. 30457571 PMC6244184

[ref31] Broeckling C. D.; Afsar F. A.; Neumann S.; Ben-Hur A.; Prenni J. E. RAMClust: A Novel Feature Clustering Method Enables Spectral-Matching-Based Annotation for Metabolomics Data. Anal. Chem. 2014, 86, 6812–6817. 10.1021/ac501530d. 24927477

[ref32] Sumner L. W.; Amberg A.; Barrett D.; Beale M.; Beger R.; Daykin C.; Fan T.; Fiehn O.; Goodacre R.; Griffin J. L.; Hankemeier T.; Hardy N.; Harnly J.; Higashi R.; Kopka J.; Lane A.; Lindon J. C.; Marriott P.; Nicholls A.; Reily M.; Thaden J.; Viant M. R. Proposed Minimum Reporting Standards for Chemical Analysis Chemical Analysis Working Group (CAWG) Metabolomics Standards Initiative (MSI). Metabolomics 2007, 3, 211–221. 10.1007/s11306-007-0082-2. 24039616 PMC3772505

[ref33] Schymanski E. L.; Jeon J.; Gulde R.; Fenner K.; Ruff M.; Singer H. P.; Hollender J. Identifying Small Molecules via High Resolution Mass Spectrometry: Communicating Confidence. Environ. Sci. Technol. 2014, 48, 2097–2098. 10.1021/es5002105. 24476540

[ref34] Kind T.; Liu K. H.; Lee D. Y.; Defelice B.; Meissen J. K.; Fiehn O. LipidBlast *in Silico* Tandem Mass Spectrometry Database for Lipid Identification. Nat. Methods 2013, 10, 755–758. 10.1038/nmeth.2551. 23817071 PMC3731409

[ref35] Smith C. A.; O’Maille G.; Want E. J.; Qin C.; Trauger S. A.; Brandon T. R.; Custodio D. E.; Abagyan R.; Siuzdak G. METLIN: A Metabolite Mass Spectral Database. Ther. Drug Monit. 2005, 27, 747–751. 10.1097/01.ftd.0000179845.53213.39. 16404815

[ref36] Horai H.; Arita M.; Kanaya S.; Nihei Y.; Ikeda T.; Suwa K.; Ojima Y.; Tanaka K.; Tanaka S.; Aoshima K.; Oda Y.; Kakazu Y.; Kusano M.; Tohge T.; Matsuda F.; Sawada Y.; Hirai M. Y.; Nakanishi H.; Ikeda K.; Akimoto N.; Maoka T.; Takahashi H.; Ara T.; Sakurai N.; Suzuki H.; Shibata D.; Neumann S.; Iida T.; Tanaka K.; Funatsu K.; Matsuura F.; Soga T.; Taguchi R.; Saito K.; Nishioka T. MassBank: A Public Repository for Sharing Mass Spectral Data for Life Sciences. J. Mass Spectrom. 2010, 45, 703–714. 10.1002/jms.1777. 20623627

[ref37] Han X. Lipidomics for Studying Metabolism. Nat. Rev. Endocrinol. 2016, 12, 668–679. 10.1038/nrendo.2016.98. 27469345

[ref38] Giles C.; Takechi R.; Lam V.; Dhaliwal S. S.; Mamo J. C. L. Contemporary Lipidomic Analytics: Opportunities and Pitfalls. Prog. Lipid Res. 2018, 71, 86–100. 10.1016/j.plipres.2018.06.003. 29959947

[ref39] Greer J. B.; Magnuson J. T.; Hester K.; Giroux M.; Pope C.; Anderson T.; Liu J.; Dang V.; Denslow N. D.; Schlenk D. Effects of Chlorpyrifos on Cholinesterase and Serine Lipase Activities and Lipid Metabolism in Brains of Rainbow Trout (*Oncorhynchus mykiss*). Toxicol. Sci. 2019, 172, 146–154. 10.1093/toxsci/kfz167. PMC6813751 31359069

[ref40] Olsvik P. A.; Søfteland L. Mixture Toxicity of Chlorpyrifos-Methyl, Pirimiphos-Methyl, and Nonylphenol in Atlantic Salmon (*Salmo salar*) Hepatocytes. Toxicol. Rep. 2020, 7, 547–558. 10.1016/j.toxrep.2020.03.008. 32373476 PMC7191540

[ref41] Olsvik P. A.; Berntssen M. H. G.; Søfteland L. Modifying Effects of Vitamin E on Chlorpyrifos Toxicity in Atlantic Salmon. PLoS One 2015, 10, e0119250 10.1371/journal.pone.0119250. 25774794 PMC4361336

[ref42] Olsvik P. A.; Hammer S. K.; Sanden M.; Søfteland L. Chlorpyrifos-Induced Dysfunction of Lipid Metabolism Is Not Restored by Supplementation of Polyunsaturated Fatty Acids EPA and ARA in Atlantic Salmon Liver Cells. Toxicol. In Vitro 2019, 61, 104655 10.1016/j.tiv.2019.104655. 31536758

[ref43] Xiang D.; Chu T.; Li M.; Wang Q.; Zhu G. Effects of Pyrethroid Pesticide *Cis*-Bifenthrin on Lipogenesis in Hepatic Cell Line. Chemosphere 2018, 201, 840–849. 10.1016/j.chemosphere.2018.03.009. 29554630

[ref44] Olsvik P. A.; Berntssen M. H. G.; Søfteland L.; Sanden M. Transcriptional Effects of Dietary Chlorpyrifos-methyl Exposure in Atlantic Salmon (*Salmo salar*) Brain and Liver. Comp. Biochem. Physiol., Part D: Genomics Proteomics 2019, 29, 43–54. 10.1016/j.cbd.2018.11.003. 30419482

[ref45] Yang J. S.; Qi W.; Farias-Pereira R.; Choi S.; Clark J. M.; Kim D.; Park Y. Permethrin and Ivermectin Modulate Lipid Metabolism in Steatosis-Induced HepG2 Hepatocyte. Food Chem. Toxicol. 2019, 125, 595–604. 10.1016/j.fct.2019.02.005. 30738135 PMC6527113

[ref46] Berland C.; Montalban E.; Perrin E.; Di Miceli M.; Nakamura Y.; Martinat M.; Sullivan M.; Davis X. S.; Shenasa M. A.; Martin C.; Tolu S.; Marti F.; Caille S.; Castel J.; Perez S.; Salinas C. G.; Morel C.; Hecksher-Sørensen J.; Cador M.; Fioramonti X.; Tschöp M. H.; Layé S.; Venance L.; Faure P.; Hnasko T. S.; Small D. M.; Gangarossa G.; Luquet S. H. Circulating Triglycerides Gate Dopamine-Associated Behaviors through DRD2-Expressing Neurons. Cell Metab. 2020, 31, 773.e11–790.e11. 10.1016/j.cmet.2020.02.010. 32142669 PMC7250662

[ref47] Berland C.; Cansell C.; Hnasko T. S.; Magnan C.; Luquet S. Dietary Triglycerides as Signaling Molecules That Influence Reward and Motivation. Curr. Opin. Behav. Sci. 2016, 9, 126–135. 10.1016/j.cobeha.2016.03.005. 28191490 PMC5295493

[ref48] Paoletti L.; Elena C.; Domizi P.; Banchio C. Role of Phosphatidylcholine during Neuronal Differentiation. IUBMB Life 2011, 63, 714–720. 10.1002/iub.521. 21818839

[ref49] van der Veen J. N.; Kennelly J. P.; Wan S.; Vance J. E.; Vance D. E.; Jacobs R. L. The Critical Role of Phosphatidylcholine and Phosphatidylethanolamine Metabolism in Health and Disease. Biochim. Biophys. Acta, Biomembr. 2017, 1859, 1558–1572. 10.1016/j.bbamem.2017.04.006. 28411170

[ref50] Tocher D. R. Glycerophospholipid Metabolism. In Biochemistry and Molecular Biology of Fishes; Hochachka P. W.; Mommsen T. P., Eds.; Elsevier: Amsterdam, 1995; Vol. 4.

[ref51] Cui Z.; Houweling M. Phosphatidylcholine and Cell Death. Biochim. Biophys. Acta, Mol. Cell Biol. Lipids 2002, 1585, 87–96. 10.1016/S1388-1981(02)00328-1. 12531541

[ref52] Exton J. H. Phosphatidylcholine Breakdown and Signal Transduction. Biochim. Biophys. Acta, Lipids Lipid Metab. 1994, 1212, 26–42. 10.1016/0005-2760(94)90186-4. 8155724

[ref53] Billah M. M.; Anthes J. C. The Regulation and Cellular Functions of Phosphatidylcholine Hydrolysis. Biochem. J. 1990, 269, 281–291. 10.1042/bj2690281. 2201284 PMC1131573

[ref54] Farooqui A. A.; Horrocks L. A.; Farooqui T. Glycerophospholipids in Brain: Their Metabolism, Incorporation into Membranes, Functions, and Involvement in Neurological Disorders. Chem. Phys. Lipids 2000, 106, 1–29. 10.1016/S0009-3084(00)00128-6. 10878232

[ref55] Wright M. M.; Howe A. G.; Zaremberg V. Cell Membranes and Apoptosis: Role of Cardiolipin, Phosphatidylcholine, and Anticancer Lipid Analogues. Biochem. Cell Biol. 2004, 82, 18–26. 10.1139/o03-092. 15052325

[ref56] Whiley L.; Sen A.; Heaton J.; Proitsi P.; et al. Evidence of Altered Phosphatidylcholine Metabolism in Alzheimer’s Disease. Neurobiol. Aging 2014, 35, 271–278. 10.1016/j.neurobiolaging.2013.08.001. 24041970 PMC5866043

[ref57] Li Z.; Vance D. E. Thematic Review Series: Phosphatidylcholine and Choline Homeostasis. J. Lipid Res. 2008, 49, 1187–1194. 10.1194/jlr.R700019-JLR200. 18204095

[ref58] Gargouri B.; Bhatia H. S.; Bouchard M.; Fiebich B. L.; Fetoui H. Inflammatory and Oxidative Mechanisms Potentiate Bifenthrin-Induced Neurological Alterations and Anxiety-like Behavior in Adult Rats. Toxicol. Lett. 2018, 294, 73–86. 10.1016/j.toxlet.2018.05.020. 29775722

[ref59] Ullah S.; Ahmad S.; Altaf Y.; Dawar F. U.; Anjum S. I.; Baig M. M. F. A.; Fahad S.; Al-Misned F.; Atique U.; Guo X.; Nabi G.; Wanghe K. Bifenthrin Induced Toxicity in *Ctenopharyngodon idella* at an Acute Concentration: A Multi-Biomarkers Based Study. J. King Saud Univ., Sci. 2022, 34, 101752 10.1016/j.jksus.2021.101752.

[ref60] Rickman O. J.; Baple E. L.; Crosby A. H. Lipid Metabolic Pathways Converge in Motor Neuron Degenerative Diseases. Brain 2020, 143, 1073–1087. 10.1093/brain/awz382. 31848577 PMC7174042

[ref61] Tracey T. J.; Steyn F. J.; Wolvetang E. J.; Ngo S. T. Neuronal Lipid Metabolism: Multiple Pathways Driving Functional Outcomes in Health and Disease. Front. Mol. Neurosci. 2018, 11, 10 10.3389/fnmol.2018.00010. 29410613 PMC5787076

[ref62] Sanden M.; Olsvik P. A.; Søfteland L.; Rasinger J. D.; Rosenlund G.; Garlito B.; Ibáñez M.; Berntssen M. H. G. Dietary Pesticide Chlorpyrifos-Methyl Affects Arachidonic Acid Metabolism Including Phospholipid Remodeling in Atlantic Salmon (*Salmo salar* L.). Aquaculture 2018, 484, 1–12. 10.1016/j.aquaculture.2017.10.033.

[ref63] Segarra A.; Mauduit F.; Amer N. R.; Biefel F.; Hladik M. L.; Connon R. E.; Brander S. M. Salinity Changes the Dynamics of Pyrethroid Toxicity in Terms of Behavioral Effects on Newly Hatched Delta Smelt Larvae. Toxics 2021, 9, 40 10.3390/toxics9020040. 33672739 PMC7924609

[ref64] Mundy P. C.; Carte M. F.; Brander S. M.; Hung T. C.; Fangue N.; Connon R. E. Bifenthrin Exposure Causes Hyperactivity in Early Larval Stages of an Endangered Fish Species at Concentrations That Occur during Their Hatching Season. Aquat. Toxicol. 2020, 228, 105611 10.1016/j.aquatox.2020.105611. 32949974 PMC7938764

[ref65] Frank D. F.; Brander S. M.; Hasenbein S.; Harvey D. J.; Lein P. J.; Geist J.; Connon R. E. Developmental Exposure to Environmentally Relevant Concentrations of Bifenthrin Alters Transcription of MTOR and Ryanodine Receptor-Dependent Signaling Molecules and Impairs Predator Avoidance Behavior across Early Life Stages in Inland Silversides (*Menidia beryllina*). Aquat. Toxicol. 2019, 206, 1–13. 10.1016/j.aquatox.2018.10.014. 30414561 PMC6464817

[ref66] Li Q.; Wang M.; Tan L.; Wang C.; Ma J.; Li N.; Li Y.; Xu G.; Li J. Docosahexaenoic Acid Changes Lipid Composition and Interleukin-2 Receptor Signaling in Membrane Rafts. J. Lipid Res. 2005, 46, 1904–1913. 10.1194/jlr.M500033-JLR200. 15930520

[ref67] Simons K.; Toomre D. Lipid Rafts and Signal Transduction. Nat. Rev. Mol. Cell Biol. 2000, 1, 31–41. 10.1038/35036052. 11413487

[ref68] Varshney P.; Yadav V.; Saini N. Lipid Rafts in Immune Signalling: Current Progress and Future Perspective. Immunology 2016, 149, 13–24. 10.1111/imm.12617. 27153983 PMC4981613

[ref69] Head B. P.; Patel H. H.; Insel P. A. Interaction of Membrane/Lipid Rafts with the Cytoskeleton: Impact on Signaling and Function: Membrane/Lipid Rafts, Mediators of Cytoskeletal Arrangement and Cell Signaling. Biochim. Biophys. Acta, Biomembr. 2014, 1838, 532–545. 10.1016/j.bbamem.2013.07.018. PMC3867519 23899502

[ref70] Afoke I. K.; Igho O. E. A Histomorphologic Analysis of Pyrethroid Pesticide on the Cerebrum and Cerebellum of Adult Albino Rats. J. Exp. Clin. Anat. 2014, 13, 54 10.4103/1596-2393.154401.

[ref71] Ullah R.; Zuberi A.; Naeem M.; Ullah S. Toxicity to Hematology and Morphology of Liver, Brain and Gills during Acute Exposure of Mahseer (*Tor putitora*) to Cypermethrin. Int. J. Agric. Biol. 2015, 17, 199–204.

[ref72] Ullah S.; Li Z.; Arifeen M. Z. U.; Khan S. U.; Fahad S. Multiple Biomarkers Based Appraisal of Deltamethrin Induced Toxicity in Silver Carp (*Hypophthalmichthys molitrix*). Chemosphere 2019, 214, 519–533. 10.1016/j.chemosphere.2018.09.145. 30278404

[ref73] Muthuviveganandavel V.; Muthuraman P.; Muthu S.; Srikumar K. A Study on Low Dose Cypermethrin Induced Histopathology, Lipid Peroxidation and Marker Enzyme Changes in Male Rat. Pestic. Biochem. Physiol. 2008, 91, 12–16. 10.1016/j.pestbp.2007.11.010.

[ref74] Magnuson J. T.; Fuller N.; Huff Hartz K. E.; Anzalone S.; Whitledge G. W.; Acuña S.; Lydy M. J.; Schlenk D. Dietary Exposure to Bifenthrin and Fipronil Impacts Swimming Performance in Juvenile Chinook Salmon (*Oncorhynchus tshawytscha*). Environ. Sci. Technol. 2022, 56, 5071–5080. 10.1021/acs.est.1c06609. 35353479 PMC9354086

[ref75] Fuller N.; Magnuson J. T.; Huff Hartz K. E.; Fulton C. A.; Whitledge G. W.; Acuña S.; Schlenk D.; Lydy M. J. Effects of Dietary Cypermethrin Exposure on Swimming Performance and Expression of Lipid Homeostatic Genes in Livers of Juvenile Chinook Salmon, *Oncorhynchus tshawytscha*. Ecotoxicology 2021, 30, 257–267. 10.1007/s10646-021-02352-2. 33534069

[ref76] Fuller N.; Huff Hartz K. E.; Johanif N.; Magnuson J. T.; Robinson E. K.; Fulton C. A.; Poynton H. C.; Connon R. E.; Lydy M. J. Enhanced Trophic Transfer of Chlorpyrifos from Resistant *Hyalella azteca* to Inland Silversides (*Menidia beryllina*) and Effects on Acetylcholinesterase Activity and Swimming Performance at Varying Temperatures. Environ. Pollut. 2021, 291, 118217 10.1016/j.envpol.2021.118217. 34583267

