# Supplementary material for: The Use of Non-targeted Lipidomics and Histopathology to Characterize the Neurotoxicity of Bifenthrin to Juvenile Rainbow Trout (Oncorhynchus mykiss)
Source: Environ Sci Technol. 2022 Jul 25;56(16):11482–92. doi: 10.1021/acs.est.2c01542 (PMC9387103; doi:10.1021/acs.est.2c01542)
Supplement: Supplementary file 1 — es2c01542_si_001.pdf [file es2c01542_si_001.pdf]

The use of non-targeted lipidomics and histopathology to characterize the neurotoxicity of bifenthrin to juvenile rainbow trout (*Oncorhynchus mykiss*)

Jason T. Magnuson[*,†], Leslie Caceres[†], Nathan Sy[†], Chenyang Ji[‡], Philip Tanabe[†], Jay Gan[†], Michael J. Lydy[§], Daniel Schlenk[†,‖]

[†]Department of Environmental Sciences, University of California, Riverside, California 92521, United States

[‡]College of Environment, Zhejiang University of Technology, Hangzhou, 310032, China

[§]Department of Zoology, Center for Fisheries, Aquaculture and Aquatic Sciences, Southern Illinois University, Carbondale, Illinois 62901, United States

[‖]Institute of Environmental Health, College of Environmental and Resource Sciences, Zhejiang University, Hangzhou 310058, China

*Corresponding author:

Jason Magnuson

jason.magnuson@ucr.edu

Department of Environmental Sciences, University of California Riverside, 2460A Geology, Riverside, CA 92521, United States

Number of pages: 9
Number of figures: 13
Number of tables: 1

Table S1. Mean bifenthrin concentrations (± standard deviation) in exposure water collected throughout the two-week treatment. Mean percent recoveries are based on PCB-209 surrogate standards.

Target nominal concentration (ng/L)	Mean bifenthrin (ng/L)	Mean recovery (%)	Mean bifenthrin corrected (ng/L)
0	nd	94.3 ± 10.1	nd
30	28.3 ± 6.0	90.3 ± 10.9	31.6 ± 7.8
60	48.0 ± 12.3	78.9 ± 18.1	63.4 ± 23.9

nd- not detected

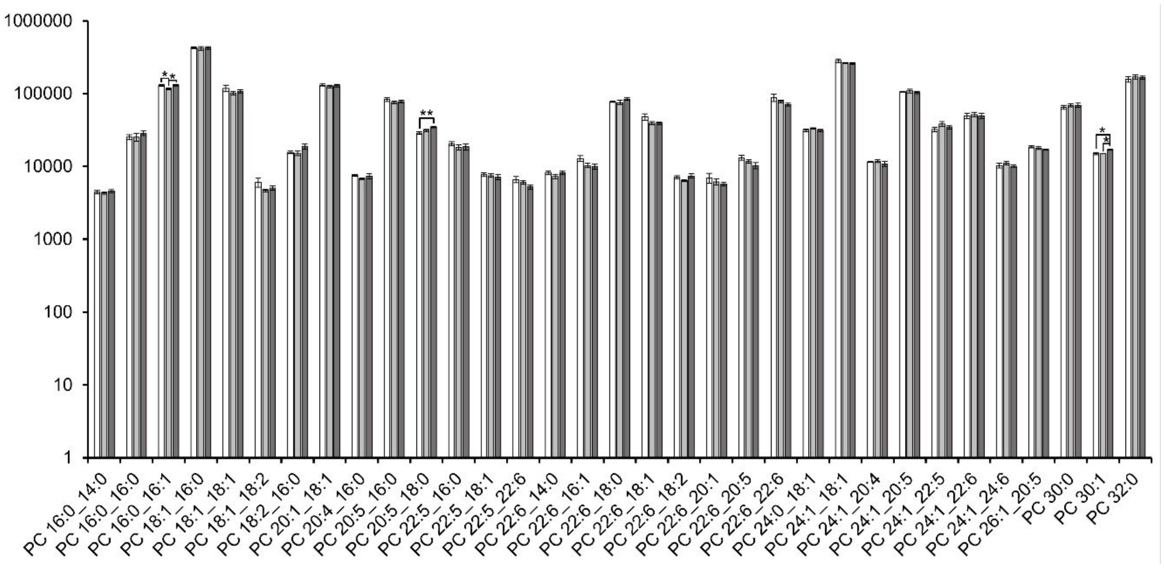

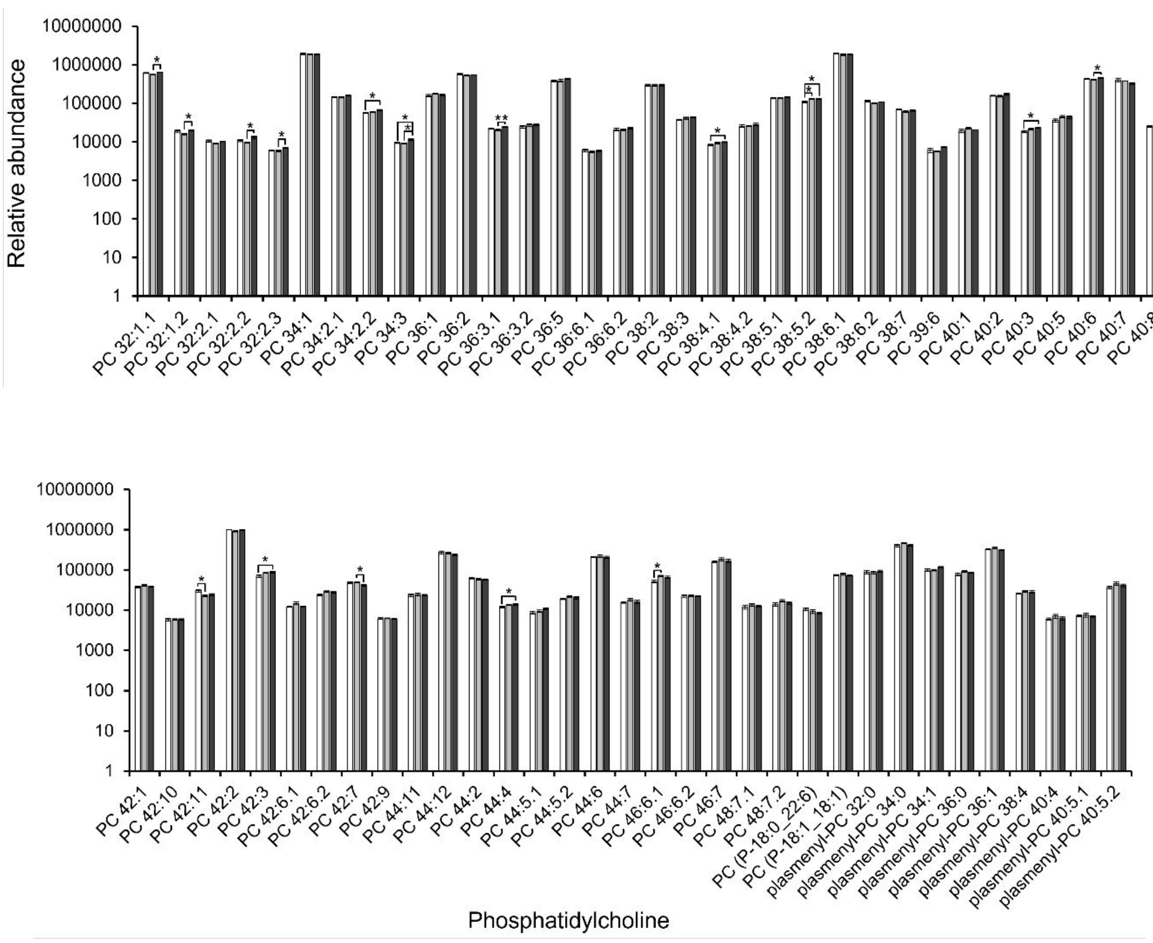

Figure S1. Mean relative abundance (± SEM) of phosphatidylcholine (PC) and plasmenyl-PC in the brains of juvenile rainbow trout treated with 0, 30, or 60 ng/L bifenthrin for two weeks. (One-way ANOVA followed by a Tukey's post-hoc; asterisks denote statistical significance- *p<0.05 and **p<0.001. n=4 for each exposure treatment).

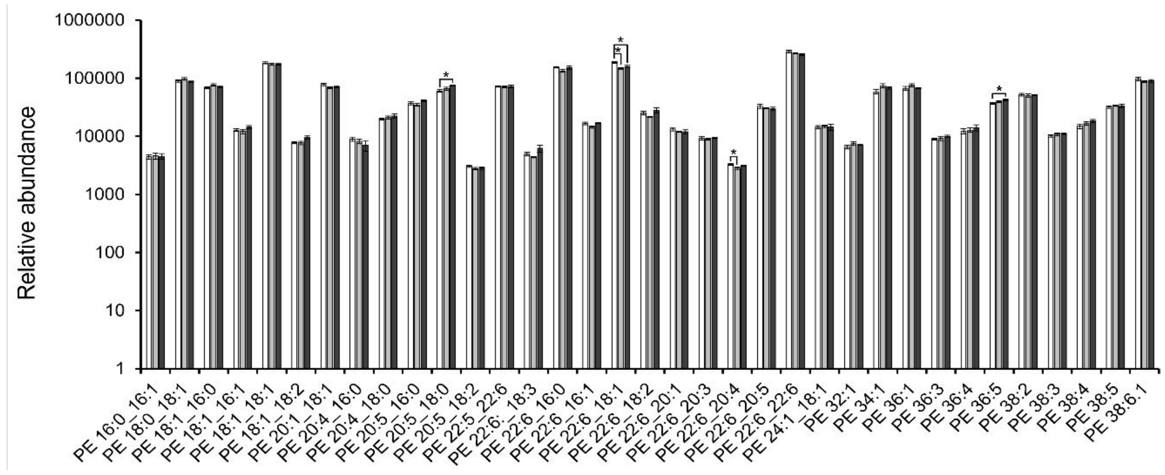

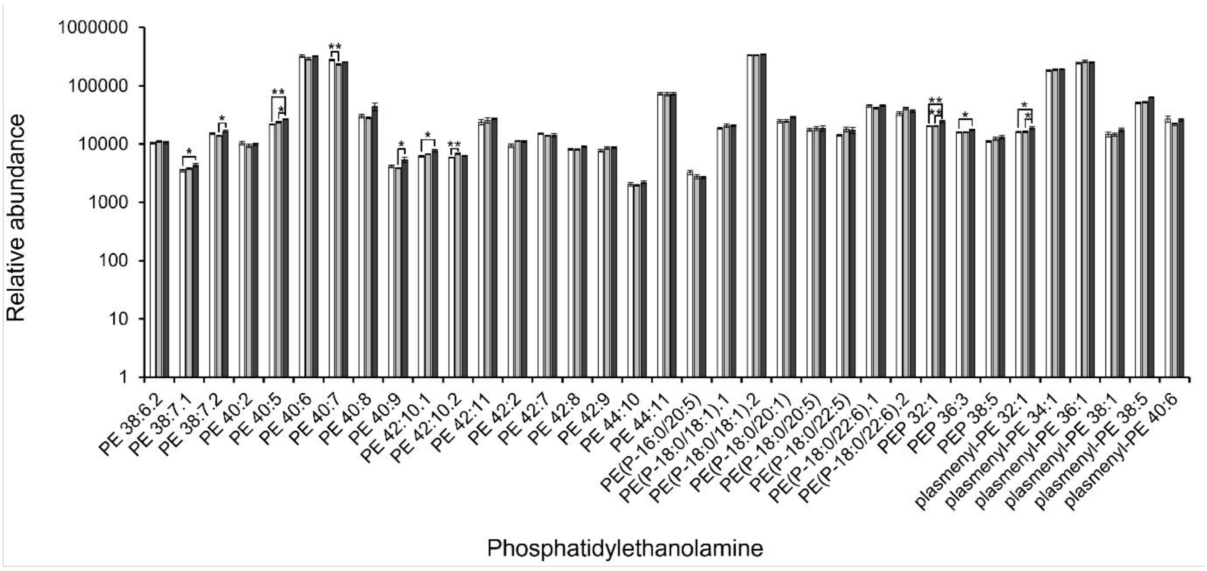

Figure S2. Mean relative abundance (± SEM) of phosphatidylethanolamine (PE) and plasmenyl-PE in the brains of juvenile rainbow trout treated with 0, 30, or 60 ng/L bifenthrin for two weeks. (One-way ANOVA followed by a Tukey's post-hoc; asterisks denote statistical significance- *p<0.05 and **p<0.001. n=4 for each exposure treatment).

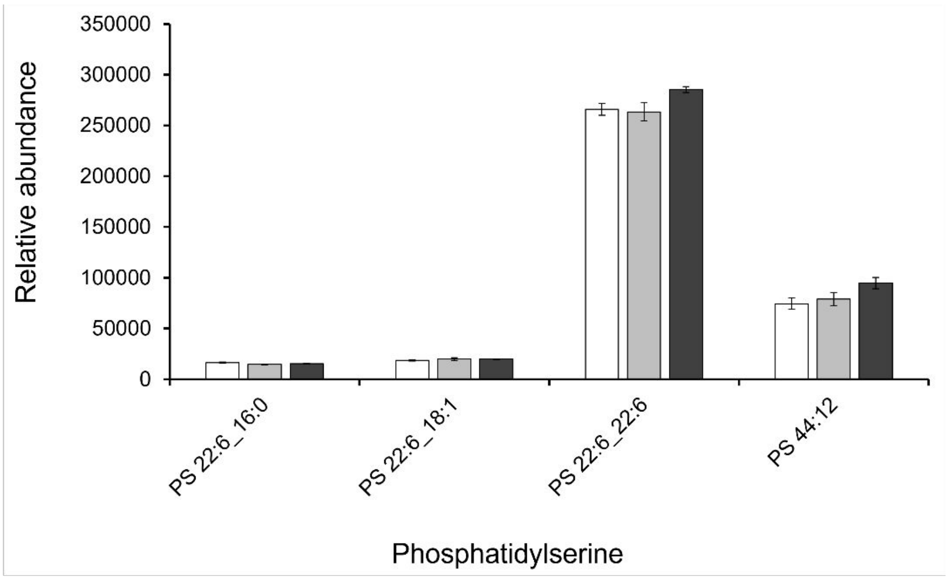

Figure S3. Mean relative abundance (± SEM) of phosphatidylserine (PS) in the brains of juvenile rainbow trout treated with 0, 30, or 60 ng/L bifenthrin for two weeks. (One-way ANOVA followed by a Tukey's post-hoc. n=4 for each exposure treatment).

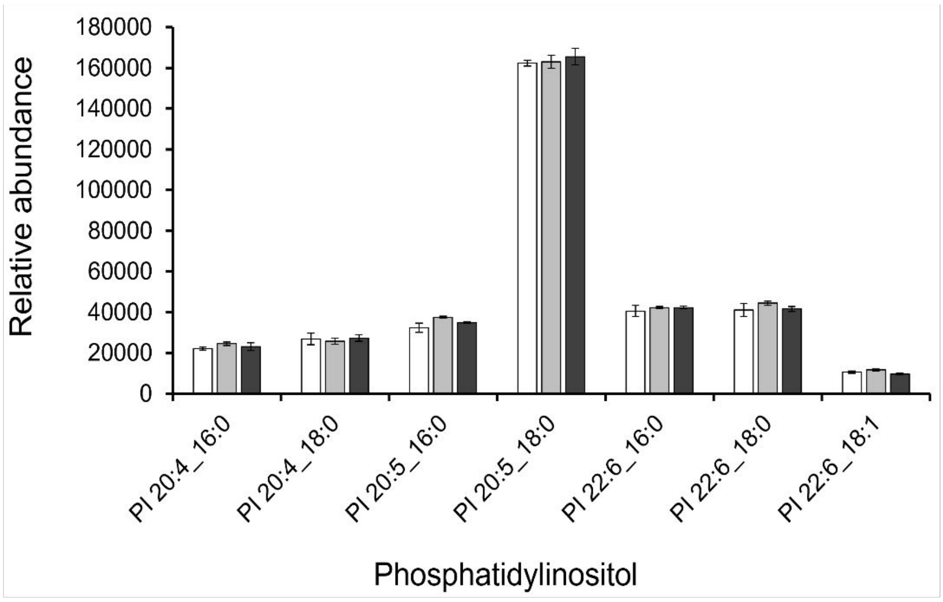

Figure S4. Mean relative abundance (± SEM) of phosphatidylinositol (PI) in the brains of juvenile rainbow trout treated with 0, 30, or 60 ng/L bifenthrin for two weeks. (One-way ANOVA followed by a Tukey's post-hoc. n=4 for each exposure treatment).

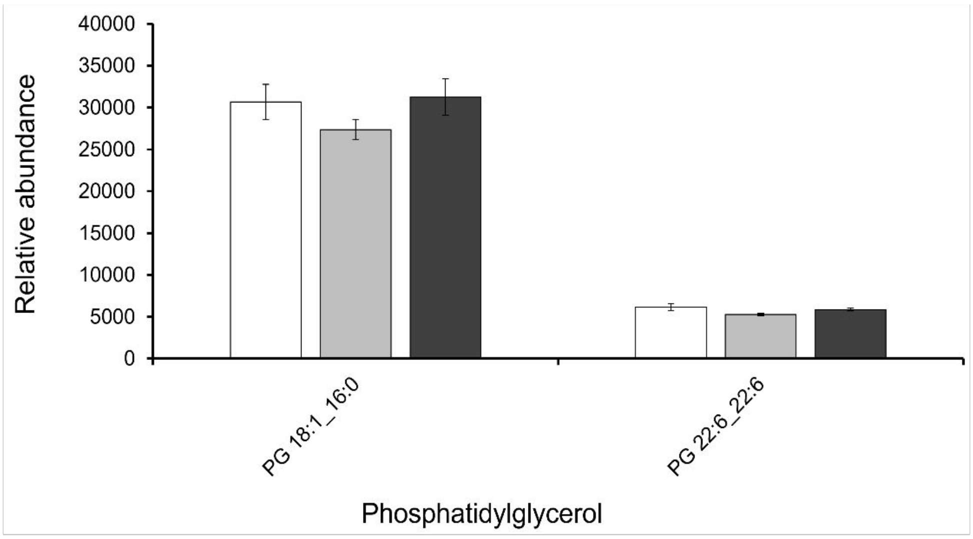

Figure S5. Mean relative abundance (± SEM) of phosphatidylglycerol (PG) in the brains of juvenile rainbow trout treated with 0, 30, or 60 ng/L bifenthrin for two weeks. (One-way ANOVA followed by a Tukey's post-hoc. n=4 for each exposure treatment).

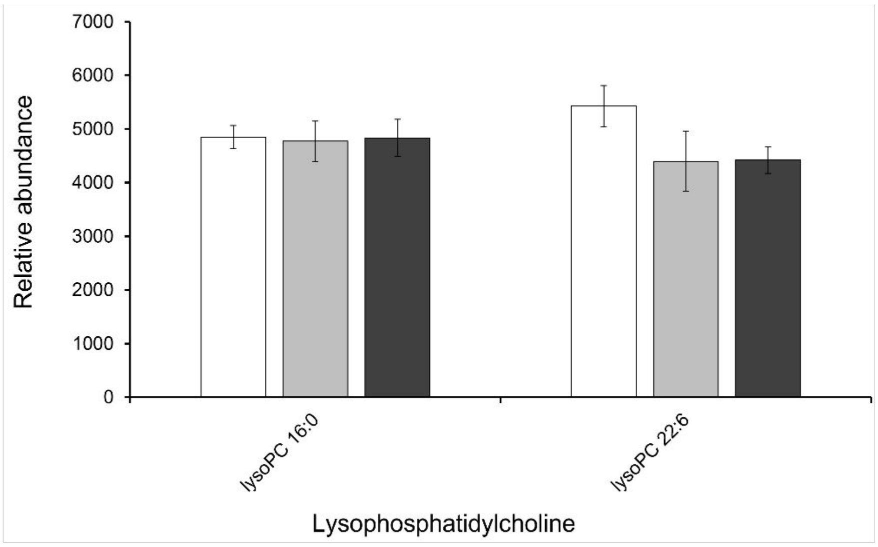

Figure S6. Mean relative abundance (± SEM) of lysophosphatidylcholine (lysoPC) in the brains of juvenile rainbow trout treated with 0, 30, or 60 ng/L bifenthrin for two weeks. (One-way ANOVA followed by a Tukey's post-hoc. n=4 for each exposure treatment).

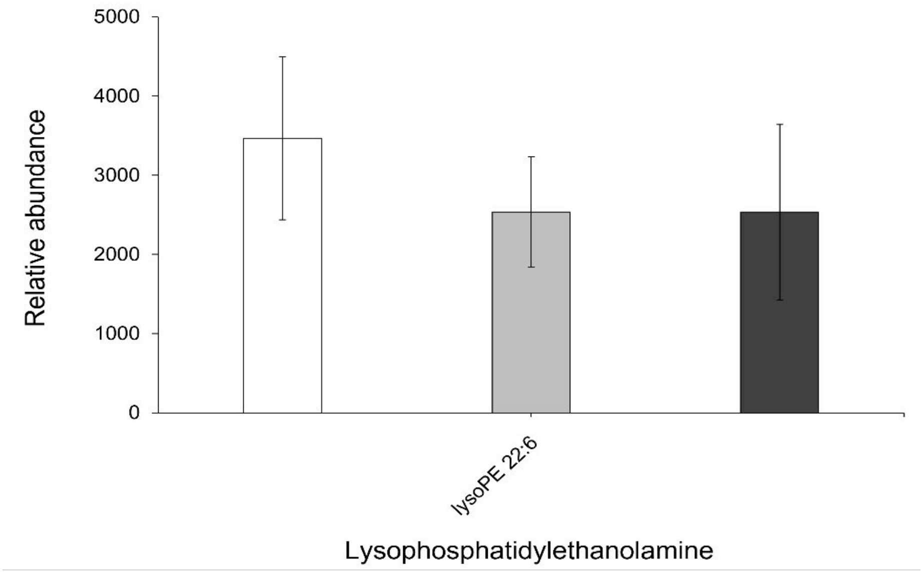

Figure S7. Mean relative abundance (± SEM) of lysophosphatidylethanolamine (lysoPE) in the brains of juvenile rainbow trout treated with 0, 30, or 60 ng/L bifenthrin for two weeks. (One-way ANOVA followed by a Tukey's post-hoc. n=4 for each exposure treatment).

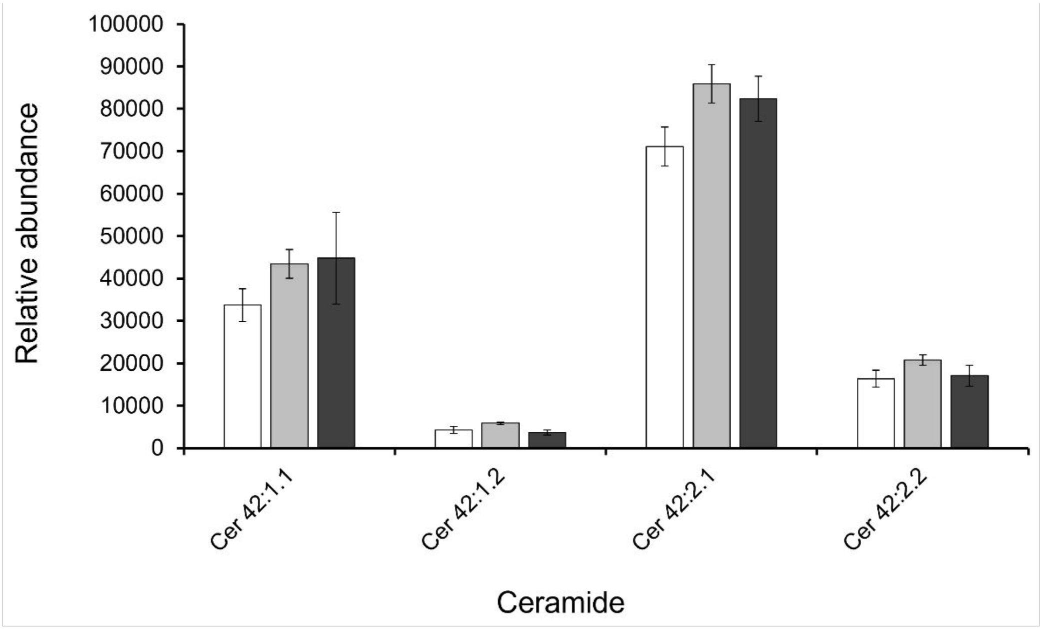

Figure S8. Mean relative abundance (± SEM) of ceramide (Cer) in the brains of juvenile rainbow trout treated with 0, 30, or 60 ng/L bifenthrin for two weeks. (One-way ANOVA followed by a Tukey's post-hoc. n=4 for each exposure treatment).

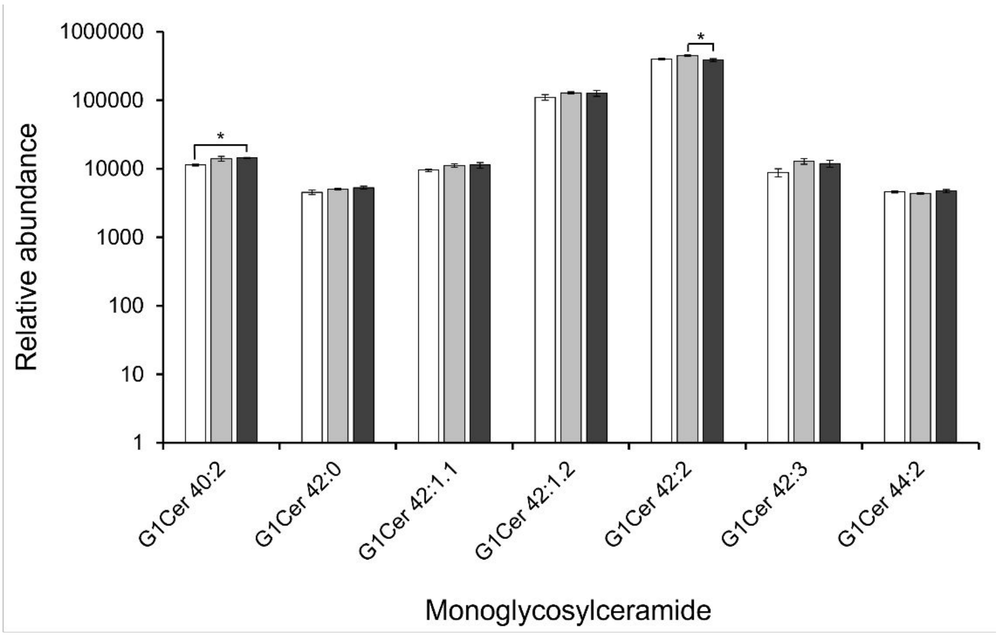

Figure S9. Mean relative abundance (± SEM) of monoglycosylceramide (G1Cer) in the brains of juvenile rainbow trout treated with 0, 30, or 60 ng/L bifenthrin for two weeks. (One-way ANOVA followed by a Tukey's post-hoc; asterisks denote statistical significance- *$p<0.05$. n=4 for each exposure treatment).

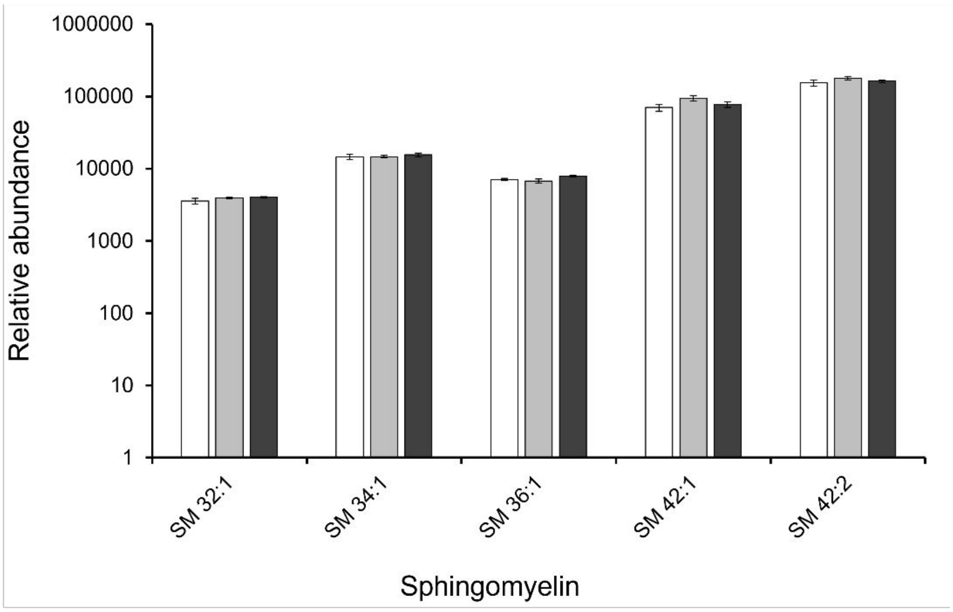

Figure S10. Mean relative abundance (± SEM) of sphingomyelin (SM) in the brains of juvenile rainbow trout treated with 0, 30, or 60 ng/L bifenthrin for two weeks. (One-way ANOVA followed by a Tukey's post-hoc. n=4 for each exposure treatment).

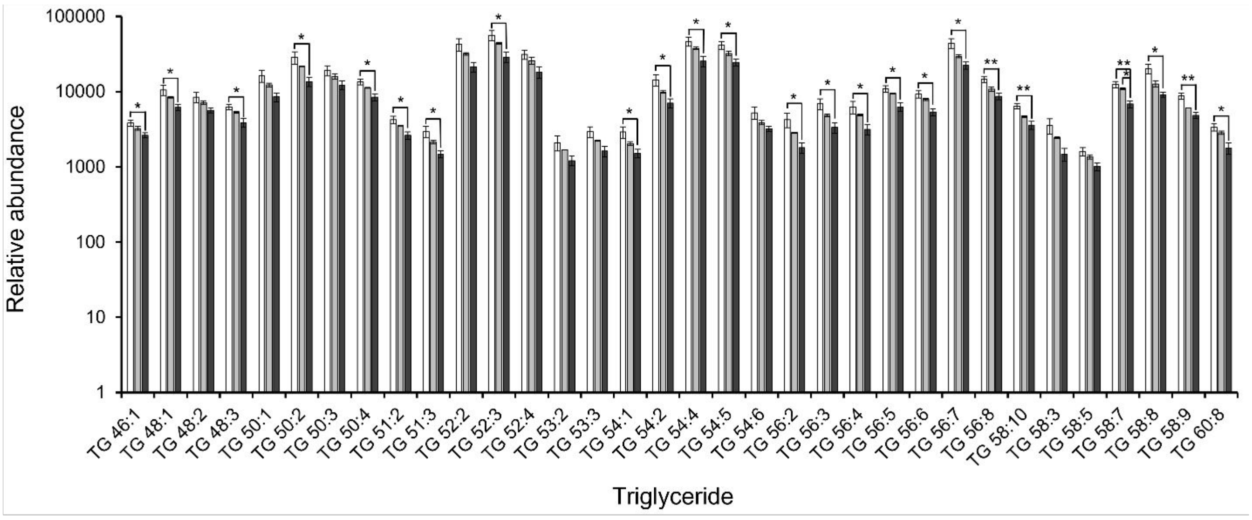

Figure S11. Mean relative abundance (± SEM) of triglycerides (TGs) in the brains of juvenile rainbow trout treated with 0, 30, or 60 ng/L bifenthrin for two weeks. (One-way ANOVA followed by a Tukey's post-hoc; asterisks denote statistical significance- *p<0.05 and **p<0.001. n=4 for each exposure treatment).

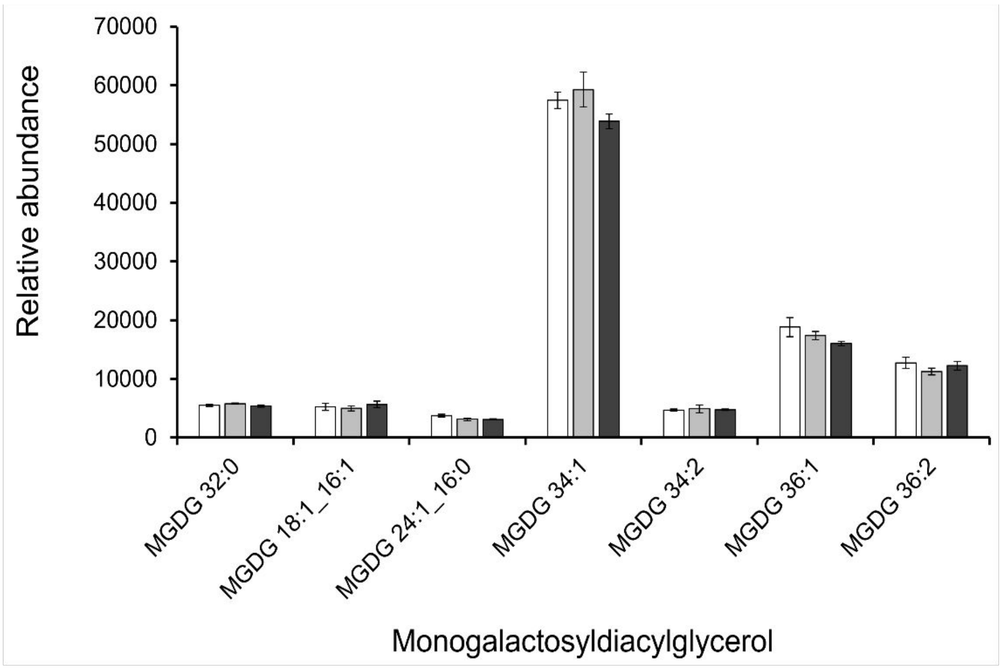

Figure S12. Mean relative abundance (± SEM) of monogalactosyldiacylglycerol (MGDG) in the brains of juvenile rainbow trout treated with 0, 30, or 60 ng/L bifenthrin for two weeks. (One-way ANOVA followed by a Tukey's post-hoc. n=4 for each exposure treatment).

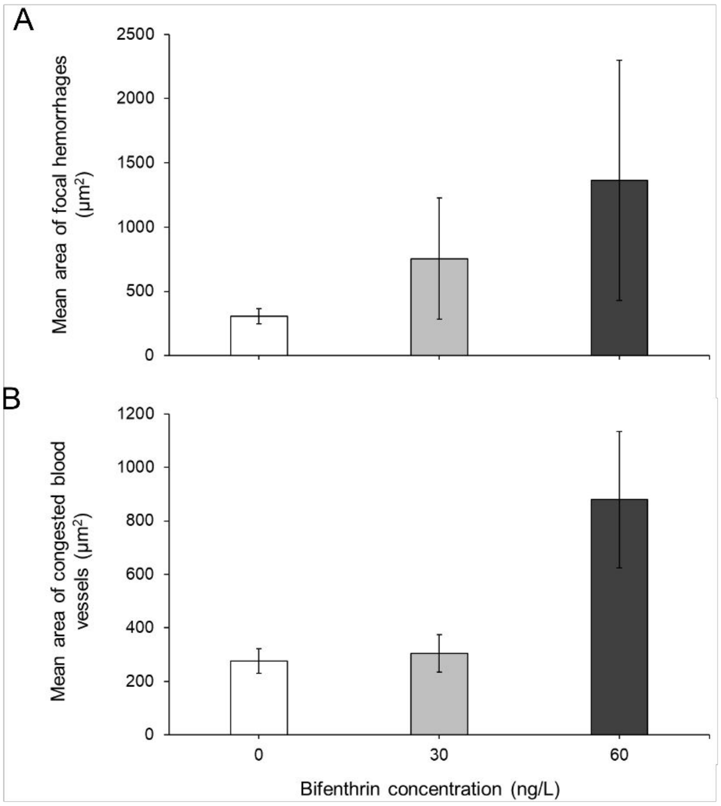

Figure S13. Mean area (± SEM) of (A) focal hemorrhages and (B) congested blood vessels in the brains of rainbow trout treated with 30 or 60 ng/L bifenthrin.
